# Lipid Cubic Systems for Sustained and Controlled Delivery of Antihistamine Drugs

**DOI:** 10.1021/acs.molpharmaceut.1c00279

**Published:** 2021-09-22

**Authors:** Michele Dully, Miriama Ceresnakova, David Murray, Tewfik Soulimane, Sarah P. Hudson

**Affiliations:** † Department of Chemical Sciences, SSPC, the Science Foundation Ireland Research Centre for Pharmaceuticals, Bernal Institute, University of Limerick, Castletroy, Co. Limerick V94 T9PX, Ireland; ‡ COOK Ireland Limited, O’Halloran Rd, Castletroy, Co. Limerick V94 N8X2, Ireland

**Keywords:** lipid cubic phase, controlled delivery, hydrophobic active pharmaceuticals, antihistamines, mucoadhesion, SAXS

## Abstract

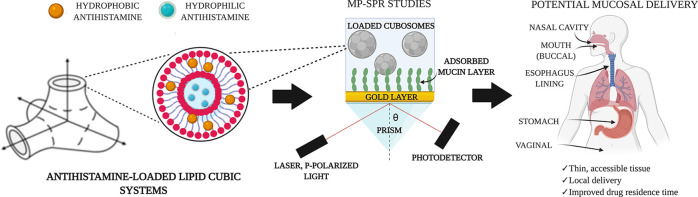

Antihistamines are capable of blocking mediator responses in allergic reactions including allergic rhinitis and dermatological reactions. By incorporating various H_1_ receptor antagonists into a lipid cubic phase network, these active ingredients can be delivered locally over an extended period of time owing to the mucoadhesive nature of the system. Local delivery can avoid inducing unwanted side effects, often observed after systematic delivery. Lipid-based antihistamine delivery systems are shown here to exhibit prolonged release capabilities. In vitro drug dissolution studies investigated the extent and release rate of two model first-generation and two model second-generation H_1_ antagonist antihistamine drugs from two monoacyglycerol-derived lipid models. To optimize the formulation approach, the systems were characterized macroscopically and microscopically by small-angle X-ray scattering and polarized light to ascertain the mesophase accessed upon an incorporation of antihistamines of varying solubilities and size. The impact of encapsulating the antihistamine molecules on the degree of mucoadhesivity of the lipid cubic systems was investigated using multiparametric surface plasmon resonance. With the ultimate goal of developing therapies for the treatment of allergic reactions, the ability of the formulations to inhibit mediator release utilizing RBL-2H3 mast cells with the propensity to release histamine upon induction was explored, demonstrating no interference from the lipid excipient on the effectiveness of the antihistamine molecules.

## 1 Introduction

Histamine, a biogenic amine whose synthesis in tissue mast cells is driven by the decarboxylation of the free amino acid histidine,^1^ is released in mammals in an inflammatory response to tissue injury or allergic reactions through a complex cascade of mediator release and interactions.^1^ Should an imbalance between accumulated histamine and the rate of its degradation occur, histamine intolerance induces a number of unwelcome side effects^2,3^ including skin wheals and itchy flare-ups through direct contact, ingestion, or inhalation of allergens.^4−6^ In allergic rhinitis, which is purported to affect over one-third of the world’s population,^7,8^ symptoms such as itching, watery eyes, and rhinorrhea are induced. Currently, the primary course of treatment for managing such allergies is oral dosage forms of antihistamines that target the histamine receptors present on the various cells in the body, of which four have been identified: H_1–4_. Of the four, H_1_ and H_2_ receptors are currently the most clinically relevant when it comes to treating histamine-related disorders. H_1_ is a receptor present on endothelial and smooth muscle cells that is the target of the majority of marketed and identified antihistamine molecules. More than 45 H_1_-antihistamines are commercially available^9^ and are referred to as inverse agonists,^10^ which bind H_1_ receptors without effecting a response, to inhibit the action of histamine through a competitive or pharmacological antagonism.^11^ They have also proven their ability to inhibit mast cell activation and subsequent histamine release, likely through the downregulation of calcium ions in the cell, although the mechanism is still not fully understood.^9,12−14^ These H_1_-antihistamines are further classified into two groups based on their ability to cross the blood-brain barrier. First-generation antihistamines are lipophilic in nature, have relatively low molecular weight, and lack of recognition by the P-glycoprotein efflux pump; they readily cross this barrier and interact with H_1_ receptors through the central nervous system (CNS).^12,15−17^ Because of a lack of selectivity, this class of molecules may also induce deleterious effects such as sedation and a reduced psychomotor performance.^18^ To overcome these, less lipophilic second-generation antihistamines have been developed that bind more specifically to H_1_ receptors and display a strong affinity for surface P-glycoprotein expressed on vascular endothelial cells reducing the likelihood of their penetration into the CNS.^18,19^

**Figure 1:**
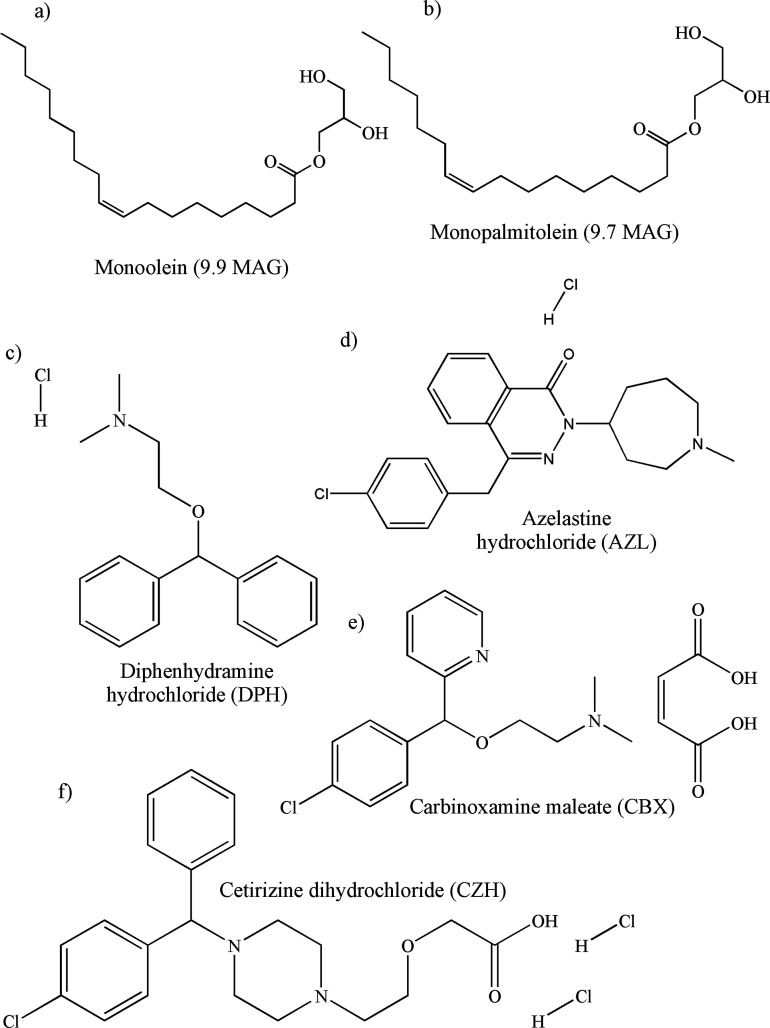
Chemical structures of (a) monoolein (9.9 MAG) and (b) monopalmitolein (9.7 MAG) both possessing an ester linkage linking the oleic acid chain to the glycerol backbone and the antihistamine drugs (c) DPH, p*K*_a_ 8.76;^20^ (d) AZL, p*K*_a_ 8.87;^21^ (e) CBX, p*K*_a_ 8.88;^22^ and (f) cetirizine dihydrochloride (CZH), p*K*_a_ 8.00.^23^

Because of their clinical relevance, a panel of first- and second-generation H_1_ receptor blockers varying in structure and solubility have been chosen for investigation. Their different physicochemical properties, described in detail in Table S1, will impact their interactions with, and subsequently their rate of release from, the selected mucoadhesive lipid-based delivery system discussed below. The key physiochemical properties, indications, and commercialized administration routes and formulations associated with the four selected antihistamines investigated have also been summarized in Table S1, and their chemical structures are shown in Figure 1.

For the most part, these inverse agonists are delivered through oral dosage forms; however, this nonspecific delivery may induce side effects including nausea, dry mouth, drowsiness, and sedation.^24,25^ To overcome these effects, a local delivery to the skin or nasal passage for atopic/contact allergic reactions would confine the effect to the delivery area, while still delivering effective concentrations to the affected organ for sustained action. Numerous drug delivery systems (DDS) have been described in the literature in the realm of effective antihistamine delivery^6,26,27^ including: nasal delivery through chitosan-derived microspheres;^28−33^ oral delivery by ethosomes,^29^ liposomes,^31^ poly(4-methyl-1-pentene), ethylene-vinyl acetate membranes and matrices;^32^ and surfactants.^34^ However, the requirement for harmful and carcinogenic excipients such as plasticizers in the formulation approach,^32^ poor stability,^35−38^ and the low encapsulation efficacy, sometimes as low as 33%,^29^ leave room for the development of more effective delivery systems. Further, the selected antihistamine molecules function in a concentration-dependent manner, where a sufficient concentration of the antihistamine molecule must be maintained over the required time to effectively compete with histamine. Although therapeutically effective, the various DDS formulations described above demonstrated rapid release (<24 h), with some reporting the peak plasma concentration after only 2 or 8 h followed by a rapid decline in concentration.^31^ The use of a controlled-release drug carrier system is therefore an attractive approach to improving the efficacy of these molecules.

In the last 20 years, lipid systems have found themselves under extensive investigation as an alternative DDS to previously applied polymeric systems in the application of drug delivery.^39,40^ One particular lipid system, the lipid cubic phase (LCP), possesses a number of physicochemical properties that make it an ideal candidate for the delivery of active pharmaceutical ingredients. The phase itself is formed under defined conditions of temperature and aqueous concentration,^41,42^ an effect that is driven by the desire of amphiphilic lipid molecules to minimize the aqueous exposure of their hydrophobic moieties through a self-assembled arrangement where their polar head-groups are oriented toward the aqueous environment.^43^ The LCP may present an advantageous approach over a traditional nonspecific systemic delivery of such antihistamine molecules, especially those first-generation and poorly soluble H_1_ antagonists that are highly lipophilic in nature and can induce unwanted side effects. The mucoadhesive nature of the system could serve to improve the local retention time of the formulation to allow for a sustained release.^44^ Not only that, but LCP systems have been shown to enhance a transdermal permeation of drugs,^45^ while the precursor lipids display an inherent biocompatibility.^46,47^ The formulations are themselves nonallergenic with a low toxicity of their digestive products.^48^ Further, the uncomplicated manufacturing requirements of the systems, which generally do not necessitate the use of organic solvents,^49,50^ mean production costs may be reduced. These features, coupled with its thermostability and resistance against dilution, make the cubic phase and its dispersions appealing for drug delivery applications.^51,52^

The cubic phase has previously been investigated for its capabilities in transdermal and ocular delivery of active ingredients as a topically applied formulation^53−55^ and has been shown to enhance the transdermal permeation of drugs such as diclofenac sodium formulated in cubic systems of glyceryl monooleate.^45^ This may be particularly relevant for cetirizine dichloride (CZH), one of the antihistamines investigated here, as it has proven effective in dermatological and nasal treatments.^11,56^ The nose has previously been investigated as an entry route for the delivery of odorranalectin-loaded cubosomes, as a more direct route to the blood-brain barrier demonstrating an enhanced therapeutic effect.^57^ However, little has been done in the way of a topical lipid delivery system to be applied on the mucosal lining on the surface of the inner nasal cavity. The aim of this investigation was to study the potential of the cubic phase, in its bulk and dispersed form, for use in the controlled delivery of various commercially available antihistamine molecules for potential use as a topical/local or more controlled oral delivery system. In vitro drug dissolution was applied to study the extent and release rate of two model first-generation and two model second-generation H_1_ antagonist antihistamine drugs from two monoacyglycerol-derived models (Figure 1). To optimize the formulation approach, the systems were characterized by small-angle X-ray scattering (SAXS) to ascertain the mesophase accessed upon incorporation of the antihistamines of varying solubilities and size. The impact of encapsulating the antihistamine molecules on the mucoadhesivity of the lipid cubic systems was also investigated using multiparametric surface plasmon resonance (MP-SPR). Facilitated by a model cell system, the internalization and associated cytotoxicity of the dispersed cubic forms are discussed. With the ultimate goal of developing therapies for the treatment of allergic reactions, the ability of the formulations to inhibit histamine release from RBL-2H3 mast cells was explored.

## 2 Material and Methods

### Materials

All solvents were of an analytical grade and purchased from FisherScientific; Monoolein 9.9 MAG (1-(9*Z*-octadecenoyl)-rac-glycerol) and monopalmitolein 9.7 MAG (1-(9*Z*-hexadecenoyl)-rac-glycerol) were acquired from JenaBioscience at greater than 99% purity. Phosphate-buffered saline (PBS) tablets were purchased from Merck. Fasted state simulated gastric fluid (FaSSGF) powder was purchased from Biorelevant.com Ltd. Water was purified in the lab using a Milli-Q Water System (Millipore Corporation). Lipase isolated from porcine pancreas was purchased from Merck (Type II, 100–500 units/mg protein (using olive oil (30 min incubation)). Cetirizine dihydrochloride, azelastine hydrochloride, carbinoxamine maleate, and diphenhydramine hydrochloride were purchased from Merck at greater than or equal to 98% purity; 3-(4,5-dimethylthiazol-2-yl)-2,5-diphenyltetrazolium bromide (MTT) Thiazolyl Blue Tetrazolium Bromide (M5655), CaCl_2_, SDS (75746), KCl, CHAPS detergent, ammonia, HCl (320331), hydrogen peroxide, glucose, mucin from bovine submaxillary gland (M3895), Dulbecco’s Modified Eagle’s Medium (D5796), and fetal bovine serum (F7524), were all purchased from Merck. Rabbit monoclonal [RM122] to IgE, rabbit IgG monoclonal [EPR25A]–isotype control, native human IgE protein (Azide free), and the Histamine ELISA kit used were purchased from Abcam; RBL-2H3 rat basophilic leukemia cells (ATCC-CRL-2256) and Eagle’s minimum essential medium (EMEM) were purchased from ATCC.

### Preparation of the Bulk Antihistamine-LCP Matrix Formulations

Two different approaches were taken in the preparation of lipid cubic formulations, depending on the solubility of the antihistamine molecule to be encapsulated in its network. For the preparation of LCP containing the water-soluble antihistamines (diphenhydramine hydrochloride (DPH) and carbinoxamine maleate (CBX)), the active pharmaceutical ingredients (APIs) were first dissolved in water (100 or 80 mg in 4 mL of water for monoolein (MO) or monopalmitolein (MPL) formulations, respectively) with sonication to ensure a complete dissolution. The fusion of dry lipid crystals to the melt was achieved between 40 and 45 °C in an oven to allow for a more facile delivery of the lipid to sample vials. Appropriate volumes of molten lipid (MO 60 mg/sample or MPL 50 mg/sample) were added to glass vials. The API-water mixture was then added to molten lipid in appropriate concentrations (40 and 50 μL of antihistamine stock for MO and MPL, respectively) to access the lipid cubic phase and to deliver API at a concentration of 1 mg per 100 mg of hydrated gel. The API-water mixture acted as the aqueous phase (≥40 wt % and ≥50 wt % for MO and MPL LCP, respectively) according to their respective phase diagrams.^1−3^ A different approach was taken to reconstitute the more hydrophobic antihistamine molecules azelastine hydrochloride (AZL) and cetirizine dihydrochloride (CZH) into LCP. The antihistamines were added (1 mg added to every 60 mg of MO or 50 mg of MPL, to give a final drug concentration of 1 mg for 100 mg of gel total) to the molten lipid prior to an addition of Milli-Q water (40 μL and 50 μL for MO and MPL, respectively) acting as the aqueous phase. The samples were then subjected to vortex mixing for no less than 15 min. The homogeneous mixtures were stored in sealed glass vials and allowed to equilibrate in the dark for at least 48 h.

The preparation of blank gels followed the same approach, without the addition of the respective drugs to the lipid/aqueous phase.

### Preparation of Cubic Dispersions (Cubosomes)

The method for the preparation of the cubosomes in this study followed that of Boge et al. with some minor modification.^58^ MO LCP was formulated with the various antihistamines at a loading concentration of 1 wt % as described previously, before being subjected to fragmentation. The preloaded/blank (antihistamine-free) bulk gel (500 mg) was added to 20 mL of 1 wt % stabilizer Pluronic F-127 solution prepared in PBS. A fragmentation was achieved by subjecting the LCP-stabilizer mixture to mixing using a magnetic stir bar followed by a high shear homogenization at 14 000 rpm using a T25 digital ULTRA-TURRAX disperser (IKA-Werke GmbH & Co. KG) for 2 min. The samples were subjected to a further fragmentation with an ATPIO ultrasonic microwave combined reaction system sonication probe operating at 40% of its maximum power on pulse mode (3 s pulses followed by a 7 s break) for an additional 5 min. The resultant milky dispersions were stored in sealed glass vials. Antihistamine-loaded cubic phases were also dispersed in the absence of stabilizer to assess changes in the physical properties of the systems.

### SAXS Investigations

LCP samples were prepared as described above with or without antihistamines and analyzed by small-angle X-ray scattering (SAXS). SAXS measurements were performed within 24 h of sample preparation at the Solution State SAXS B21 beamline at Diamond Light Source on the Harwell Campus, Didcot, UK, as previously described.^59^ Samples were stored in sealed vials until just before the data acquisition to avoid any sample dehydration through an atmospheric exposure. The experiments used a beam of wavelength λ = 13.1 keV (∼0.946 44 Å) with a beam size at the sample of 1 mm × 1 mm. The data collection was performed at ambient temperature (20 °C). B21 utilizes a bending magnet source with a typical flux of ∼4 × 10^12^ photons per second delivered directly to the sample. The photons were distributed over a large 0.8 × 2 mm cross-section that served to minimize radiation damage while also enhancing the signal of the particles. Two-dimensional (2D) diffraction images were recorded on an Eiger X 4 M detector, with a detector face size of 155.2 mm × 162.5 mm and pixel size of 75 μm × 75 μm. The beam size at the detector was 50 μm × 50 μm. The detector was configured to measure a scattering vector (q) range from 0.0032 to 0.38 Å^–1^. Bulk LCP samples were loaded into a custom three-dimensional (3D) printed sample holder designed for viscous samples. The holder was printed in 3D from a mixture of methacrylic acid esters and photoinitiator comprising a window in which the sample was filled. The sample holder was made from stainless steel and had mica windows. Each dispersed cubosome sample was carefully aspirated into a glass capillary before it was placed in the path of the beam. Each sample was subjected to a 1 s X-ray exposure for 15 frames at one location and required manual loading.

Small-angle diffraction images were processed, and the relative positions of the distinct Bragg peaks were indexed and used to deduce the space groups and lattice parameters by correlating with Miller indices as already described.^60^ Similarly, the water channel diameter and lipid chain length were calculated as previously described,^59^ with surface area and Euler Poincare constant (χ) used^61,62^ to track any changes induced upon drug loading.

### Mucoadhesion Studies

The mucoadhesion/bioadhesion of the cubosomes was evaluated using surface plasmon resonance (SPR; SPR Navi 200, BioNavis) in a similar manner to that described previously to investigate the mucoadhesive properties of block copolymer micelles.^63^ Data were collected by instrument scientists at Bionavis, Tampere, Finland. Mucin-coated sensors were prepared using mucin from a bovine submaxillary gland (M3895 SigmaAldrich). The coating was prepared according to the protocol proposed by Prosperi-Porta et al.^63^ In brief, bare Au sensors for MP-SPR measurements were first cleaned using 1:1:5 (v/v) solution of H_2_O_2_/NH_3_/H_2_O (10 min, 90 °C). After a thorough rinsing in Milli-Q water and drying under nitrogen stream, the sensors were incubated in 100 μg/mL mucin solution for 24 h at room temperature in the dark. A mucin deposition was performed and measured in situ by MP-SPR (Supporting Information). On the basis of the registered full SPR curves (not shown), the LayerSolver software by BioNavis enabled a calculation of the optical thickness of the layers formed on the sensor surface. The calculated layer thickness was 3.88 nm (±0.11) resulting in a surface coverage of 2.8 ng/mm^2^ and a refractive index of 1.41 (±0.002) at 670 nm, which is typical for a glycoprotein layer. After the mucin adsorption, the sensors were rinsed in water to remove any unbound residual mucin, and sensors were stored dry until use.

Tests were performed using the MP-SPR 2-channel Navi 220A NAALI system equipped with two detection wavelengths (670 and 785 nm) with instrument injection loops set to 1000 μL volume. Samples were run at 37 °C in PBS (pH 7.4) as the running buffer. Empty cubosome test samples were added to the PBS (pH 7.4) at a concentration of ∼15 mg gel/mL. The flow rate was set to 20 μL/min for an injection of cubosome samples. Before the samples were loaded, and in between tests, the sensor surface was preconditioned with short washes in 3-[(3-cholamidopropyl)dimethylammonio]-1-propanesulfonate (CHAPS) detergent (20 mM, 1 min injection) at a flow rate of 50 μL/min followed by a 10 min baseline stabilization with the PBS buffer. Samples at a concentration of 1 mg/mL were subsequently loaded over a 40 min injection time to reach a steady-state signal followed by 40 min of PBS buffer flow to assess the dissociation phase. Samples were loaded in at least three repetitions, with each injection separated by a rejuvenation of the sensors by a wash with CHAPS 20 mM solution (2 × 1 min injections at 50 μL/min). CHAPS provided sufficient surface regeneration and stable baselines between the samples.

### Particle Size Estimation and Zeta-Potential Studies

Dynamic light scattering was utilized to estimate the particle size distribution (Z-average) and polydispersity (PDI) as well as the zeta potential (mV) of the cubic dispersions using PBS as the dispersant on a Zetasizer (Malvern Panalytical) equipped with a 4 mW He–Ne laser (633 nm), which applies Brownian motion theories in its measurement. The viscosity of the dispersant was set at 0.8872 cP, and the system was maintained at 25 °C. Three measurements of 50 runs were taken for each sample, and the mean value, along with the calculated standard deviation (SD) for particle size estimation (nm), were recorded using the Malvern Panalytical zetasizer software.

### Powder X-ray Diffraction (PXRD)

PXRD data were collected in reflection mode with an Empyrean diffractometer (PANalytical, Phillips) equipped with Cu Kα1,2 radiation (γ = 1.5406 Å) operating at 40 kV and 40 mA at room temperature. Samples were scanned between 2θ values of 5 and 40° at a step size of 0.013 13° 2θ/s, 73 s per step.

### Encapsulation Efficacy

High-performance liquid chromatography (HPLC) was used to quantify the encapsulation efficacy (EE%) of the antihistamine-loaded cubosomes. Freshly made cubosomes were removed from the dispersion media by centrifugation at 10 000 *g* for 30 min. The concentration of free drug in the supernatant was then quantified by means of the chromatographic approach described below. The encapsulated drug could then be calculated as a percentage of the total added drug according to the following equation.
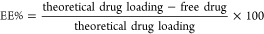
1

### Drug Release Studies

Different buffers were investigated to study the release profiles of the four antihistamines from the LCP under different conditions of pH. FaSSGF was prepared by dissolving preprepared simulated intestinal fluid (SIF) powder (Biorelevant.com) in an acidic buffer (2 g NaCl in 1 L water) and adjusting the pH to 1.6 using 1 M HCl with stirring according to the manufacturer’s guidelines. Simulated nasal fluid (SNF) was prepared as previously described by Farid et al.^64^ (7.45 mg/mL NaCl; 1.29 mg/mL KCl; 0.32 mg/mL CaCl_2_.2H_2_O; made up to volume with deionized water) to give a solution pH of 6.4. The final buffer was phosphate-buffered saline, prepared at pH 7.4.

The solubility of the four antihistamines were determined in the different release media studied. Excess amounts of each drug were added to 10 mL of media at 37 °C with shaking at 150 rpm overnight. Undissolved drug was removed by filtration before the concentration of drug in each sample was determined after an appropriate dilution by HPLC under the chromatographic separation conditions specified below.

The HPLC system used in this investigation was an Agilent 1200 Infinity Series (Agilent Technologies) comprising: G1311B 1260 quaternary pump, G1329B 1260 ALS autosampler, G1316A 1260 TCC (thermostated column compartment), and a G1365D 1260 MWD VL diode-array detector. The acquired data were processed with the Agilent OpenLAB CDS software. Chromatographic separations of antihistamine-containing samples were achieved using an Agilent Poroshell 120 PFP (3 × 100 mm, 2.7 μm) column fitted with a UHPLC Poroshell 120 guard module (3 × 5 mm, 2.7 μm). The system was maintained at 21 °C with the mobile phase delivered under isocratic conditions of 0.4 mL/min. The separation conditions for each antihistamine are described in Table 1. In all cases, the mobile phase was delivered to the column at a flow rate of 0.5 mL/min. Samples were filtered through a 0.2 μm nylon filter (Fisherbrand), and 8 μL injections were made.

**Table 1:** Chromatographic Separation Conditions for the Various Antihistamines

antihistamine	mobile phase (A/B)	ratio (A/B)	Λ_max_ (nm)	retention (min)
diphenhydramine Hydrochloride (DPH)	acetonitrile: 25 mM KH_2_PO_4_ (0.1% formic acid)	25:75	210	∼4.8
cetirizine dihydrochloride (CZH)	acetonitrile: 25 mM KH_2_PO_4_ (0.1% formic acid)	25:75	230	∼14
carbinoxamine maleate (CBX)	acetonitrile: 25 mM KH_2_PO_4_ (0.1% formic acid)	20:80	260	∼4.1
azelastine hydrochloride (AZL)	acetonitrile: 50 mM KH_2_PO_4_	40:60	215	∼4.9

All in vitro drug release testing of the antihistamines from the bulk lipid formulations/dispersions was performed in triplicate at 37 ± 0.1 °C under shaking at 150 rpm. For bulk samples, over the course of the investigation at various time points (between 0 and 216 h) the entire release media was withdrawn, and the media was immediately replenished with freshly made stock. The release of the antihistamines from the cubosomal dispersions was tracked via dialysis. For this, the drug-loaded cubosomal dispersions were placed in Pur-A-Lyzer dialysis devices with a molecular weight cutoff of 6–8 kDa. The drug release was followed in 15 mL of each of the selected release media, where 1 mL of the sample media was removed at various time points (between 0 and 48 h) and immediately replenished with the same volume of fresh media. The dissolution samples were analyzed by means of a HPLC method described previously to quantify the accumulated drug in solution.

The dissolution testing of the as-received free drug was performed in parallel under the same conditions of temperature and agitation in Duran flasks containing 100 mL of the various biorelevant media under sink conditions. Two milliliter aliquots of the dissolution media were removed using preheated syringes (37 °C) at various time points (between 0 and 60 h) and immediately replaced with prewarmed fresh media. The drug concentration was determined by means of HPLC after filtration through 0.2 μm filters and after calculations took the dilution factor into consideration.

### Cell Culture Methods

NIH-3T3 cells were grown in Dulbecco’s Modified Eagle’s Medium (DMEM) (with sodium pyruvate) supplemented with 10% v/v fetal bovine serum (FBS), 1% v/v l-glutamine, and 1% penicillin-streptomycin at 37 °C in humidified air containing 5% CO_2_. RBL-2H3 cells were cultured Eagle’s Minimum Essential Medium (EMEM) (ATCC 30–2003) supplemented with 15% v/v heat-inactivated FBS and 1% penicillin-streptomycin at 37 °C in humidified air containing 5% CO_2_.

### Cell Viability Study

The cytotoxic effect of the monoolein-based cubosomes formulated with and without the four antihistamine molecules was assessed by an MTT assay of NIH-3T3 and RBL-2H3 cells. Cells were plated in 96-well plates at a seeding density of 5 × 10^3^ cells/well. Cells were allowed to reach confluence over 2 d before the experiments were performed. The cells were then treated with cubosomes with the antihistamine drugs (at a drug loading of 1% w/w) and without (blank sample) at a concentration of 100 μg/mL in each well and incubated at 37 °C for 24 and 48 h. After the incubation period, the reagent MTT was added to samples for 2 h at 37 °C. The MTT formazan crystals were then dissolved by a solubilization buffer (10% SDS in 0.01 M HCl) followed by a further incubation for 4 h at 37 °C. The absorbance was read using a multiwell microplate spectrophotometric reader at 570 nm. The cell viability was determined as the percentage of absorbance values of treated cells to absorbance values of untreated control cells. A ratio of percentage reduction of cell viability relative to that of untreated cells (the control) was used to express the obtained data. All measurements were performed in at least triplicate.

### Cellular Uptake of Cubosomal Formulations

The difference in zeta potential between mammalian cells and the drug-loaded cubosomes was utilized to track the fusion and uptake of the lipid nanoparticles over time. Mammalian fibroblast cells (NIH-3T3) were selected as the model system. Cells were seeded in six-well plates at a seeding density of 3 × 10^5^ cells/well. Cells were allowed to reach confluency before the experiments were performed. The cells were then incubated in the presence of the various antihistamine-loaded (1% w/w drug loading) and blank (no drug) MO cubosomes at a concentration of 100 μg/mL in each well for different lengths of time (0.5, 2, and 12 h). The cells were harvested after this predetermined time by trypsinization, and the cell pellets were resuspended in 1 mL of fresh DMEM media. The zeta potential of the treated cells was measured as previously described and compared against that of the untreated cells, the control group.

### Anti-immunoglobulin E (IgE)-Induced Histamine Release Studies

RBL-2H3 cells were exposed to blank and antihistamine-loaded cubosome formulations after an IgE treatment, and the histamine release was tracked in a manner similar to that described previously.^65^ Cells were seeded in 24-well plates at a seeding density of 1 × 10^6^ cells/well. Cells were allowed to reach 90% confluence overnight before treatment. After this period, the medium was aspirated away and replenished with fresh mediaum containing 0.2 μg/mL IgE. Cells were incubated in the antibody media for 1 h at 37 °C. The medium was once again aspirated away, and cells were washed with a release buffer (1 mM CaCl_2_, 40 mM NaOH, 0.1% bovine serum albumin (BSA), 119 mM NaCl, 5 mM KCl, 5.6 mM glucose, 25 mM piperazine-*N*,*N*-bis (2-ethanesulfonic acid) (PIPES), and 0.4 mM MgCl_2_). The washed cells were subsequently treated with release buffer containing 1.25 μg/mL anti-IgE along with 100 μg/mL of the blank or antihistamine-loaded cubosome formulations. Cells were incubated for a further 10 min, at which point the medium was removed and the concentration of released histamine was determined by means of a competitive histamine enzyme-linked immunosorbent assay (ELISA) kit. Samples were diluted appropriately before analysis.

## 3 Results and Discussion

### Structural Characterization of the Mesophases

There is a wide range of host lipids capable of forming the cubic phase available commercially.^66−71^ In this study, two naturally occurring monoacylglycerol (MAG) lipids, namely, MO and MPL, were selected to form cubic systems. Both host lipids are generally regarded as safe (GRAS) listed digestive products of triglycerides present in the gastrointestinal tract, and they were selected here on account of their biodegradable nature and inherent ability to maintain the cubic phase under physicological conditions.^67,72^

Cubic phases are distinguishable by their discrete crystallographic space groups. Three inverse bicontinuous cubic phases exist; primitive (Q_II_^P^), gyroid (Q_II_^G^), and double-diamond (Q_II_^D^). The MAG-water system typically accesses two types of cubic phase under equilibrium at room and body temperature depending on the level of hydration.^42^ At lower water concentrations, the gyroid or “Q_II_^G^” cubic phase is accessed, and when hydration levels are increased, the phase transitions to the more swelled and stable diamond cubic (Q_II_^D^) phase. The Q_II_^D^ phase is stable against dilution and maintains its architecture when the water content is increased further to excess levels. The SAXS data collected at Diamond Light Source in the UK was analyzed for mesophase characterization and dimensional analysis of the bulk and dispersed systems, with and without incorporated antihistamine molecules (at a loading concentration of 1 wt %) to investigate if the incorporation of the antihistamine molecules had altered the internal structure of the lipid systems. All samples, with and without the drug molecules, were found to exist in the cubic phase (Table 2 and Supporting Information). Compatible reflections in the collected data were utilized, and the absolute values were indexed to calculate the lattice parameters of bicontinuous cubic phase samples. As discussed in the introduction, CBX and DPH, being water-soluble, may preferentially reside in the water channels of the cubic phase, while the more lipophilic agents CZH and AZL may likely integrate into the lipid portion of the system.

**Table 2:** Phase Identification and Lattice Parameters of Assigned Mesophases for Bulk LCP Loaded with Different Antihistamine Drugs (1 wt %) from SAXS Experiments with Calculated Dimensional Values for Lipid Chain Length (*L*) and Water Channel Diameter (*D*_H2O_)

host lipid	API	assigned mesophase	lattice parameter (nm)	*L* (nm)	*D*_H2O_ (nm)
MO		Q_II_^D^	10.30	1.75	4.55
MO	DPH	Q_II_^D^	11.29	1.93	4.97
		Q_II_^D^	12.66	2.16	5.57
MO	CBX	Q_II_^D^	11.82	2.01	5.22
MO	CZH	Q_II_^D^	10.47	1.78	4.61
MO	AZL	Q_II_^D^	10.80	1.84	4.75
		Q_II_^D^	12.05	2.05	5.31
MPL		Q_II_^D^	11.41	1.56	5.80
MPL	DPH	Q_II_^D^	11.73	1.61	5.95
		Q_II_^D^	13.25	1.81	6.74
MPL	CBX	Q_II_^D^	11.93	1.63	6.06
		Q_II_^D^	13.47	1.84	6.84
MPL	CZH	Q_II_^D^	10.98	1.50	5.58
MPL	AZL	Q_II_^D^	11.91	1.63	6.05
		Q_II_^D^	12.99	1.78	6.60

Table 2 reports the assigned mesophases and their structural parameters for bulk antihistamine-loaded systems calculated from the collected one-dimensional (1D) and 2D SAXS patterns, where sharp Bragg reflections indicative of a long-range order were seen (shown in the Supporting Information). For all samples, a cubic phase of the crystallographic space group Q_II_^D^ was accessed, with slightly larger lattice parameters (*L*) identified than those previously reported.^73,74^ The more hydrophilic drugs DPH and CBX were expected to locate comfortably in the aqueous channels of the phases, defined as 4.55 and 5.8 nm for blank MO and MPL LCP, respectively, both of which were prepared with water only. Hydrophobic molecules have the potential to disrupt the lipid bilayer network and induce phase transitions^75,76^ by integrating into the lipid bilayer and altering the liquid crystalline structure. However, when the hydrophobic AZL and CZH antihistamines were incorporated into the lipid cubic network, Q_II_^D^ symmetries prevailed. In some cases, the calculation of two lattice parameters pertaining to Q_II_^D^ space groups of different dimensions may be indicative of the initiation of phase transitioning caused by a sample dehydration toward Q_II_^D^ + Q_II_^G^.^42^

Larger water channels were noted across all drug-loaded systems relative to the blank systems, indicating swollen lipid structures. A more pronounced effect was noted when the highly water-soluble DPH and CBX were incorporated into the aqueous conduits of the phase, with a lesser effect noted with the more hydrophobic molecules AZL and CZH. The presence of small and broad unassignable peaks in some samples (Supporting Information) could not be related to any cubic mesophase, so it is unclear whether these relate to an intermediate/transitioning phase as has previously been described in a monoolein system^77^ or possible phase transitions beyond the cubic region.^75,76^ Such intermediates exist at temperatures below 33 °C and are not accounted for in the lipidsaxs script used here.

The fully hydrated lipid cubic phase is resistant to dilution, maintaining its structural integrity even in excess water. However, when the bulk phase is fragmented, the resulting cubosomes are not stable long-term in aqueous solution because of the hydrophobic portions that are exposed at the particle surface. The introduction of amphiphilic copolymers that adsorb to the cubosome’s surface reduces the interfacial free energy between the cubic phase and the water phase while maintaining the internal nanostructure of the phase.^78^ Further, the weak electrostatic charge on the surface of the cubosomes (discussed later) lends a need for a suitable stabilizer to reduce agglomeration of the dispersions. The accumulation of Pluronic F-127 on the external surface of the cubosomes has demonstrated an enhanced steric stabilization of cubosomes in aqueous solution dispersions,^79−81^ and, in particular, those produced using monoolein, where a small amount of the stabilizer can also be incorporated into the internal labyrinth of the water channels.^82^

In this investigation, preloaded submicron particles were produced through a high-energy homogenization and sonication of the bulk phase. As the differences in release properties between MPL and MO were minor, Figure 6, monoolein alone was selected to investigate the behavior of antihistamine cubosomes, on account of its slightly more controlled release properties overall (discussed later). In a similar way to that described for the bulk systems, SAXS was employed to reveal the architecture of theses MO lipid dispersions (Table 3) of predicted cubic symmetry. A similar trend in lattice parameter changes was seen across the cubosome samples compared to the corresponding bulk formulations, where the calculated lattice parameter followed the trend CBX > DPH > AZL > CZH.

**Table 3:** Phase Identification and Lattice Parameters of Assigned Mesophases for MO Cubosomes Loaded with Different Antihistamine APIs (1 wt %) and Stabilized with Pluronic® F-127 from SAXS Experiments

host lipid	API	assigned mesophase	lattice parameter (nm)	*L* (nm)	*D*_H2O_ (nm)
MO	CBX	Q_II_^D^	10.79	1.83	4.77
MO	DPH	Q_II_^D^	10.66	1.82	4.69
MO	CZH	Q_II_^D^	9.43	1.61	4.15
MO	AZL	Q_II_^D^	10.57	1.80	4.66

A single Q_II_^D^ symmetry was assigned for the systems. The cubosomes maintained the cubic phase of the bulk systems from which they were conceived, with only minor reductions in unit cell parameter values calculated. An efficient concentration of the stabilizer can halt transitions between the Q_II_^D^ and Q_II_^G^ structures.^81,83^

### Properties of Cubic Dispersions: Zetasizer Studies and Encapsulation Efficacies

The monoolein cubosomes were loaded with the antihistamines at a theoretical concentration of 1 wt %. HPLC was used to quantify the encapsulation efficacy (Table 4) by quantifying the amount of free drug in solution after the dispersion process. It is apparent that a better association between the lipid system and drug is seen in the cases of the hydrophobic agents, where loading efficacies of greater than 93% were calculated for both AZL and CZH. Slightly lower encapsulation efficacies (87–90%) were calculated for the hydrophilic drugs, which are likely mainly present in the water channels of the cubic network with little interaction at the lipid bilayer.

**Table 4:** Properties of Antihistamine-Loaded Cubosomes with and without Pluronic® F-127 Stabilizer and with and without the Four Antihistamines (1% w/w loading) Measured within an Hour of Preparation

Pluronic F-127 stabilizer	drug	theoretical drug loading (wt %)	EE (%)	size ± SD (nm)	PDI	zeta potential (mV)
0 wt %				182.7 ± 0.1	0.24	–6.9 ± 0.2
	DPH	1	87.5 ± 1.4	301 ± 23	0.38	–11.8 ± 1.0
	CBX	1	90.5 ± 1.2	289 ± 10	0.23	–2.9 ± 1.0
	AZL	1	93.1 ± 0.7	271 ± 20	0.20	–25.9 ± 0.5
	CZH	1	94.9 ± 0.7	153 ± 12	0.29	–15.5 ± 0.2
1 wt %				133.9 ± 0.1	0.25	1.0 ± 0.1
	DPH	1	87.5 ± 1.4	135.4 ± 1.2	0.19	7.5 ± 0.3
	CBX	1	90.5 ± 1.2	140.6 ± 4.5	0.17	4.6 ± 0.1
	AZL	1	93.1 ± 0.7	140.1 ± 2.3	0.17	17.4 ± 0.0
	CZH	1	94.9 ± 0.7	147.1 ± 4.9	0.23	2.1 ± 0.2

The particle size and zeta potential of the antihistamine-loaded cubosome formulations prepared with and without stabilizer Pluronic F-127 are shown in Table 4. An evaluation of the zeta potential of the lipid nanoparticles aids in the prediction of their stability, tendency to aggregate (greater charge means stronger electrostatic repulsion between particles),^84,85^ performance, and cellular uptake in vivo. A modification of the cubosomes, through an incorporation of additives/APIs, or alterations in surface chemistry can alter the surface charge of the dispersions and, by default, their performance.

Samples prepared in the absence of Pluronic F-127 demonstrated a large variation in nanoparticle size between the different samples encapsulating the four antihistamine molecules. The zeta potential results showed that both the blank and antihistamine-loaded cubosomes produced without Pluronic F-127 carried a negative charge. This has previously been associated with trace free fatty acids contaminating commercially available MAG lipids. These may carry a negative charge when ionized and alter the overall cubosome charge upon adsorbing onto its surface.^86^ The lipids used here report purity greater than 99%, but the potential of free oleic acid is still there. The zeta potential is taken as the overall charge the cubic nanoparticles acquire in a given dispersant and is a measure of the magnitude of repulsive/attractive forces between nanoparticles and serves as an efficient indicator of the storage stability of nanoparticles. The overall zeta potential of the samples was less than 30 mV in magnitude across the board, and so the stability of the samples is likely reduced.^87,88^

The particle size and zeta potential data were also collected for samples prepared with the commonly used stabilizing additive Pluronic F-127 (Table 4). The particle sizes of all cubosomes generated were in the nanosize range. The size varied slightly among the different encapsulated antihistamine cubosomes with a slightly larger particle size ranging between ∼135 and 147 nm compared with the blank system (134 nm). All samples displayed PDI values less than 0.25 and size reductions up to 50% lower, with greatly reduced standard deviations, compared with the systems prepared without Pluronic F-127. This result is in agreement with published data.^81,89,90^ The surface charge also varied between the cubosomes loaded with different antihistamines, and in stark contrast to the negative charge recorded for the samples prepared without stabilizer, the study revealed positively charged cubosomes in the presence of Pluronic F-127 in all cases. This study identifies the positive impact of including the stabilizer on achieving cubic dispersions with the desired size range, as nanoparticles of size less than 200 nm have shown an improved cellular uptake over larger systems^91−93^ with a reduced propensity to aggregate in solution. The stability of the cubosome-stabilizer systems was confirmed over a 5 d storage period, where no change in nanoparticle size was observed. Pluronic F-127 has previously been shown in the literature to physically stabilize the colloidal cubic phase for months,^94^ so it is reasonable to predict a more lengthy stability period than the one tested here. The positive surface charge associated with the samples was predicted to further improve the cellular uptake of the drug-loaded samples, as cationic polymers form complexes and bind with the negatively charged plasma membrane of the cells to a higher degree than negatively charged or neutral molecules.^95^

### Cellular Uptake of Cubosomes

As a means of studying the time-dependent interaction and possible uptake of the formulated cubosomes by cells, changes in the zeta potential of NIH-3T3 fibroblast cells incubated with the formulations were tracked over 12 h. It has been described that positively or weakly negatively charged nanoparticles should show an enhanced cellular uptake^96,97^ on account of the negative zeta potential on the cell surface. Previous reports showed that a 12 h incubation with nanoparticles was sufficient for the cells to fully take up the dispersions; the nanoparticles were identified by an initial change and then a stabilization of the zeta potential back to that of the untreated cells after the nanoparticles were bound and subsequently internalized.^98^ In this study, the cells were incubated with MO cubosomes for 0.5, 2, and 12 h before the zeta potential was measured and compared to that of the untreated cells.

**Figure 2:**
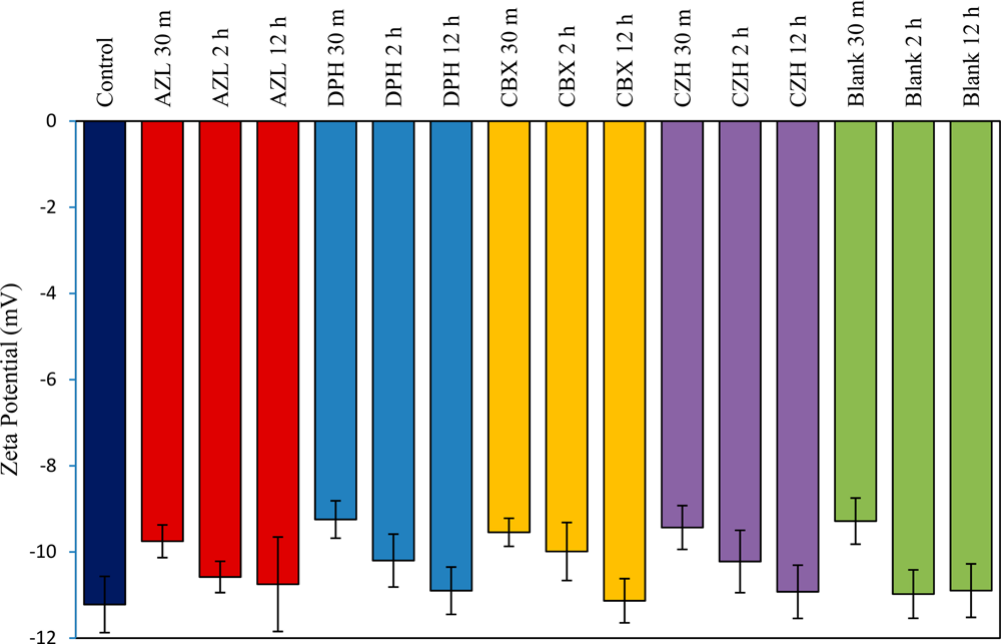
Zeta potential of NIH-3T3 cells treated with 100 μg/mL of MO cubosomes stabilized with Pluronic F-127 and formulated with antihistamine molecules (1% w/w drug loading) or blank cubosomes (no drug), measured in DMEM media after 0.5, 2, or 12 h. The control group was NIH-3T3 cells that were untreated.

Figure 2 shows the shift in zeta potential of the NIH-3T3 cells incubated with the cubosomal formulations. After 30 min, a shift toward a less negative surface charge was noted across all samples compared to that of the untreated cells. This shift may signify the binding of the positively charged cubosomes at the cell surface, reducing the overall negative charge of the cell membrane through electrostatic interactions.^99^ When nanoparticles interact and adsorb onto the cell membrane, changes in the zeta potential are seen as the adsorption of ions surrounding their surface, and the surfaces of the hydrodynamic shear and particle mobility are altered.^98^ An uptake through the cell’s plasma membrane after an adsorption can occur by means of different mechanisms including phago- or endocytosis.^98,100^ Endocytosis is the process commonly observed in the internalization of nanoparticles, soluble molecules, proteins, and lipids^101−103^ and is an energy-dependent process.^102,104,105^ The mechanism may be either nonspecific or receptor-mediated and has been widely described in the uptake of cubosomes.^106−108^ After the 12 h exposure to the cubosomes, the zeta potential returned to a more negative value and approached that of the untreated cells, suggesting that the adsorbed cubosomes could have been taken up by the cells after this time. The different cubosome samples exhibited different zeta potentials on their surfaces, from 0.97 ± 0.05 mV for the blank samples to 17.40 ± 0.01 mV with the AZL loaded samples, Table 4. Despite these differences, no significant difference in the change in zeta potential of the NIH-3T3 cells was observed between any of the cubosome treatments at any of the time points. While these alterations in zeta potential suggest the uptake of the cubosomes by the cells, the release studies, Figure 7, indicate that, over the course of this 12 h experiment, significant amounts of the antihistamines may be released prior to an internalization, which may explain the lack of differentiation between the different cubosome samples with the cells. In this case, the drugs would likely disperse in the surrounding tissue at the delivery site.^102^

### Cytotoxicity Study

Monoolein-based cubosomes formulated with each of the four studied antihistamines were evaluated for a cytotoxic effect in NIH-3T3 fibroblast cells and RBL-2H3 basophilic leukemia cells as a model by means of an MTT assay. While positively charged nanoparticles have been reported to improve drug delivery efficacy, an increase in the cytotoxic effect of these formulations has also been reported.^109^

**Figure 3:**
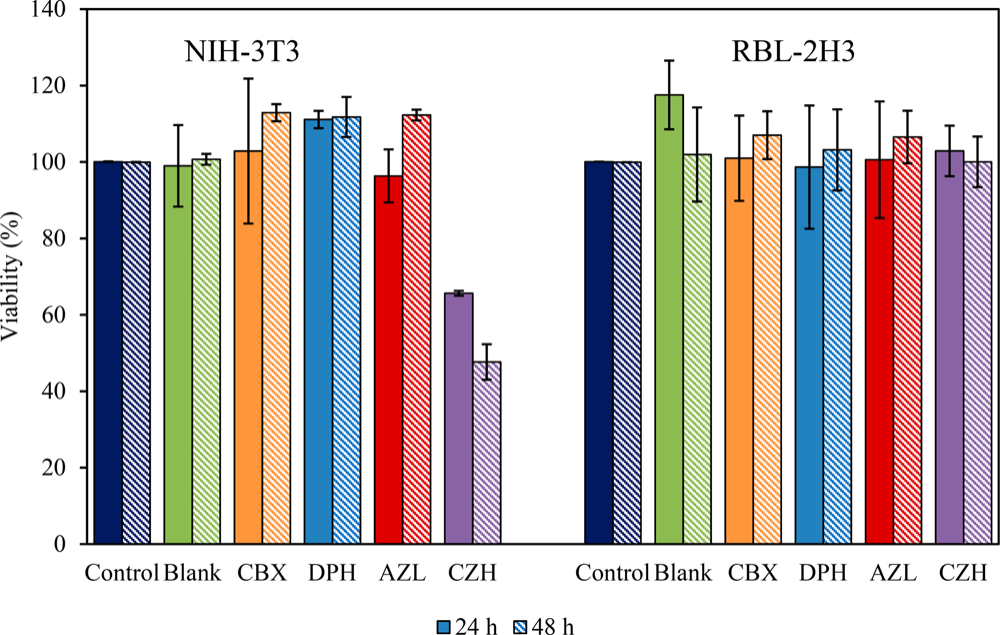
Cell viability, expressed as percentage of the control absorbance at 570 nm, induced in NIH-3T3 and RBL-2H3 cells after incubations for 24 and 48 h in the presence of MO cubosomes (delivered at ∼100 μg/mL antihistamine-loaded cubosomes, prepared at 1 wt % antihistamine loading), formulated with and without (BLK) antihistamine molecules. The control group was NIH-3T3 or RBL-2H3 cells that were untreated. Data are expressed as a mean ± SD of three independent experiments (minimum *n* = 3).

The treatment with the lipid cubic dispersions containing antihistamine molecules did not appear to significantly negatively impact the cell viability in the case of the RBL-2H3 cells, Figure 3, in agreement with published data.^110,111^ Similar results were observed in the case of the NIH-3T3 cells with the exception of those treated with CZH-loaded cubosomes when compared to the control system after 24 or 48 h, at which point ∼50–75% of the loaded drug should have been released into solution (Figure 7). Given that the CZH-loaded cubosomes had the closest zeta potential to the blank cubosomes, 2.08 ± 0.22 and 0.97 ± 0.05 mV, respectively, and no toxicity was shown for the other cubosomes with much higher zeta potentials, up to 17.40 ± 0.01 mV, the charge was not the cause of the observed cytotoxicity. The treatment with CZH at similar concentrations has previously been shown to be cytotoxic against epithelial cell lines.^112^ However, a separate study by Salimi et al. studied the cytotoxic effect of a gradient of CZH concentrations on Chang cell lines and showed that concentrations of almost 200 times more than were studied here were required to induce cytotoxicity to the degree reported here.^113^ That said, the Chang cells were only assessed over a 6 h exposure period. These published studies further serve to highlight the variation in cell line sensitivity to the antihistamine, in agreement with the differences in tolerability shown here. Further, it is possible that the cubosomes have facilitated an improved cellular uptake of the drug as reported in the literature^108,114^ and may also explain the observed increased toxic effect. Regardless, the obtained results are in agreement with literature confirming the biocompatibility of monoolein-based cubosomes,^108,115^ and the reduction in viability is likely owed to the encapsulated drugs.

### Mucoadhesion Study

The high viscosity of the in situ-formed cubic phase may facilitate its bioadhesive properties, which were demonstrated by Nielsen et al.^44^ in proposing cubic phases of glyceryl monooleate (GMO) and monolinoleate as bioadhesive mucosal drug delivery systems.^116^ In fact, the literature reports that GMO-based cubic phase gels have proven their ability to adhere to rabbit jejunum and have also been found to interact at a surface level in the vaginal cavity for a period of 6 h.^44^ A targeting of mucosal layers for a local delivery has the advantage of overcoming the first pass effect otherwise seen with enteral delivery routes to improve the bioavailability and would provide a means for a local delivery of antihistamine molecules to overcome the associated unwanted side effects of these molecules when systemically administered (especially first-generation H_1_ antihistamines).^117−121^

Here, MP-SPR was used to investigate the mucoadhesive nature of the antihistamine-cubosomes by studying their interaction with a mucin protein-coated surface using blank monoolein cubosomes as a positive control for a mucoadhesive comparison. Mucin is a highly glycosylated polymeric protein excreted^122^ by goblet and submucosal glands and constitutes between 2 and 5% of the composition of the protective mucosal layer.^123^ The mucin used would be expected to have a negative charge in PBS, as mucins tend to be rich in aspartates and glutamates, with p*K*_a_ values of 3.9 and 4.1, respectively,^124^ which should interact electrostatically with the positively charged cubosomes, Table 4. The highly entangled network of the mucin fibers is responsible for the sticky adhesive nature of the mucus^125^ and provide a means for modeling the adhesive nature of our systems in vitro. The results obtained from this in vitro mucoadhesion investigation provide an estimate of the cubosome residence time at a given mucosal site of administration.^126^

**Figure 4:**
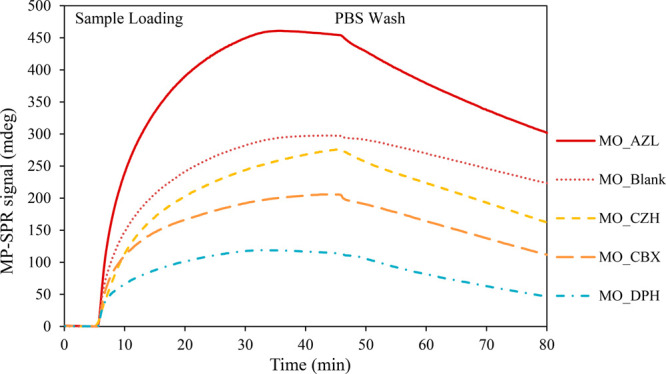
Kinetics of adsorption onto mucin-coated sensors measured for five tested cubosome formulations with antihistamine drugs (1% w/w drug loading) and without (blank). Each sensogram is the average MP-SPR signal from triplicate sample injections measured at 670 nm.

The cubosomes formulated with or without antihistamine molecules exhibited reproducible adsorption kinetic profiles as observed from triplicate injections of the dispersed samples over mucin-coated sensors (Supporting Information). Figure 4 represents an overlay of average sensograms registered for each sample. Equilibrium is reached within 40 min of injection. The data presented quite distinct behavior between the different cubosomal formulations toward the mucin layer. The MO_AZL samples displayed the highest binding level at a constant concentration of 1 mg/mL even when compared to the reference MO_Blank (unloaded cubosomes) suggesting greater mucoadhesion. The MO samples loaded with DPH displayed a much lower binding capacity to mucin—displaying an equilibrium state signal that is threefold lower than the strongly bound MO_AZL sample. Compared to the MO_blank sample, the MO_CBX formulation also displayed reduced binding, and, despite a slower association, the MO_CZH could be considered to have a similar binding efficiency to the blank unloaded cubosomes. Differences in the zeta potential were also thought to play a part in the variations in mucoadhesion, as the oligosaccharide chains of the mucin glycoprotein confer a negative charge to the protein through carboxyl and sulfate groups.^127^ The AZL system displayed the strongest positive charge, which also represented the strongest binding. On the basis of the surface charge, the mucoadhesivity trend was expected to follow according to AZL > DPH > CBX > CZH > blank cubosomes. The loading of the cubosomes onto the mucin was conducted over 40 min. From the cubosome release studies, Figure 7, the amount of antihistamine released in 40 min was approximately less than 5% for DPH, AZL, and CZH and ∼20% of CBX. Thus, the properties of the cubosomes and their surface charge would not have changed significantly over the course of the experiment. Despite this, it appears that the DPH system displayed the lowest degree of binding and that CZH cubosomes were second in line to the AZL system. On the basis of these findings, the differences may be attributed to the nature and effect of the encapsulated antihistamine molecules themselves, with the hydrophilic molecules seemingly causing a greater reduction in binding when compared to the blank system. This may be due to the additional bonding and hydrophobic interactions between the lipophilic drugs and hydrophobic segments on the mucin glycoprotein.

Despite a higher binding intensity observed in the case of the AZL-loaded cubosomes, a faster rate of dissociation indicated by the slope of the reduction in relative intensity after the washing step was noted compared to the other formulations. This decline in intensity can be taken as representative of the stability of the adsorbed layer.^128^ Regardless, the mucoadhesive nature demonstrated for these formulations supports their potential to prolong the retention of an encapsulated drug molecule at the target site for improved bioavailability and is comparable to the mucoadhesion of other DDS including pectins^129^ and hyaluronic acid-coated niosomes.^130^ An adhesion of the systems to the mucosal layer increases the drug residence time for absorption that might otherwise be cleared through a mucociliary clearance.^118,131−133^

### Drug Release Studies

The solubility of the antihistamine molecules at 37 °C was studied in different biorelevant media, and the variations in solubility at different pH are shown, Table 5. There is an obvious increase in solubility of the drugs as the pH becomes more acidic from PBS at 7.4 to SNF at pH 6.4 and, further, to FaSSGF at pH 1.6. This trend is seen across all of the antihistamine molecules as determined by HPLC. This may be related to their associated p*K*_a_ values (Table 5). AZL,^21^ DPH,^20^ and CBX^22^ are weak bases and under acidic conditions are protonated and in turn are more polar thus increasing their solubility at a lower pH. This trend is shown in Table 5, where decreasing the pH toward a more acidic environment increases the saturated solubilities of the drugs. CZH is considered a weak acid^23^ but under acidic conditions is considered to be zwitterionic.

**Table 5:** Saturated Solubility of the Model Antihistamine Drugs in a Range of Biorelevant Media, Phosphate Buffered Saline (PBS), Simulated Nasal Fluid (SNF), and Fasted State Gastric Fluid (FaSSGF) at 37 °C

antihistamine	p*K*_a_ values	solubility in PBS buffer pH ≈ 7.4 (mg/mL)	solubility in SNF buffer pH ≈ 6.4 (mg/mL)	solubility in FaSSGF buffer pH ≈ 1.6 (mg/mL)
diphenhydramine hydrochloride (DPH)	8.76 (weak base^20^)	888 ± 13	976 ± 11	1212 ± 28
carbinoxamine maleate (CBX)	8.88 (weak base^22^)	320 ± 21	514 ± 36	760 ± 10
cetirizine dihydrochloride (CZH)	2.19, 2.93, and 8.00 (weak acid^23^)	4.60 ± 0.02	6.9 ± 0.5	11.0 ± 0.1
azelastine hydrochloride (AZL)	8.87 (weak base^21^)	1.33 ± 0.01	1.40 ± 0.02	6.30 ± 0.04

On the basis of these marked variations in solubility, it was hypothesized that the pH of the dissolution medium would likely influence the release rates of the antihistamines into the dissolution medium. In vitro release data of the H_1_ receptor antagonists into biorelevant media were obtained over a two-week period and quantified using HPLC.

**Figure 5:**
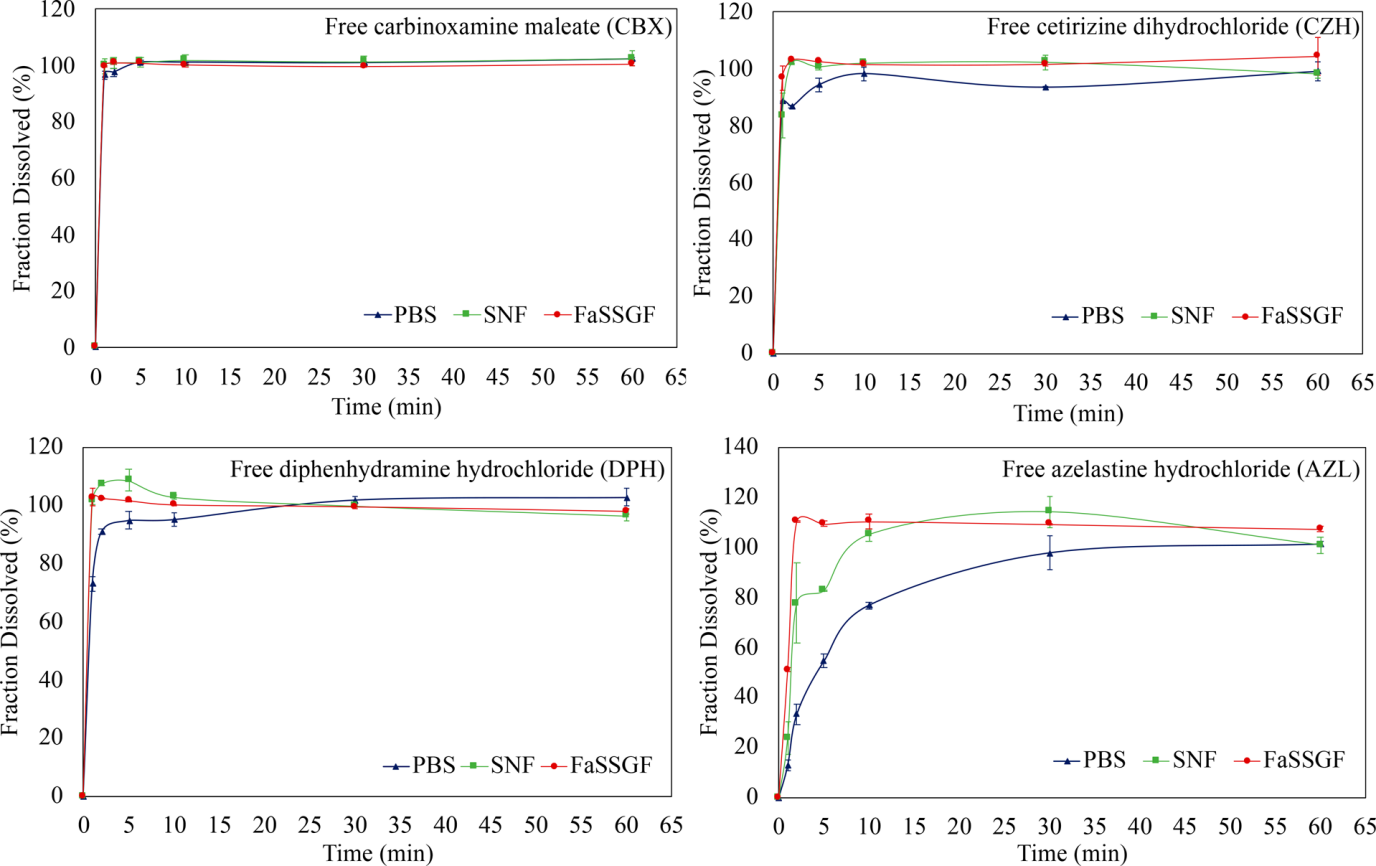
In vitro dissolution profiles of as-received (free) hydrophilic (left) and hydrophobic (right) antihistamine drug at 37 °C in PBS at pH ≈ 7.4, SNF at pH 6.4, and FaSSGF at pH ≈ 1.6. Each point represents the mean (±SD) of two determinations. The lines drawn on the plots are only to aid the reader in following the progression of the dissolution.

The as-received drugs were all crystalline (PXRD diffractogram, Supporting Information) and in agreement with structures reported in the literature.^134−137^ They all rapidly dissolved, under sink conditions, in all three aqueous media (Figure 5). In most cases, complete solubilization was recorded within the first 10 min, with only AZL showing a more prolonged release as the pH increased due to its hydrophobic nature and lower solubility. Even so, almost 80% of the AZL was in solution after the first 10 min and 100% was solvated within the first hour.

**Figure 6:**
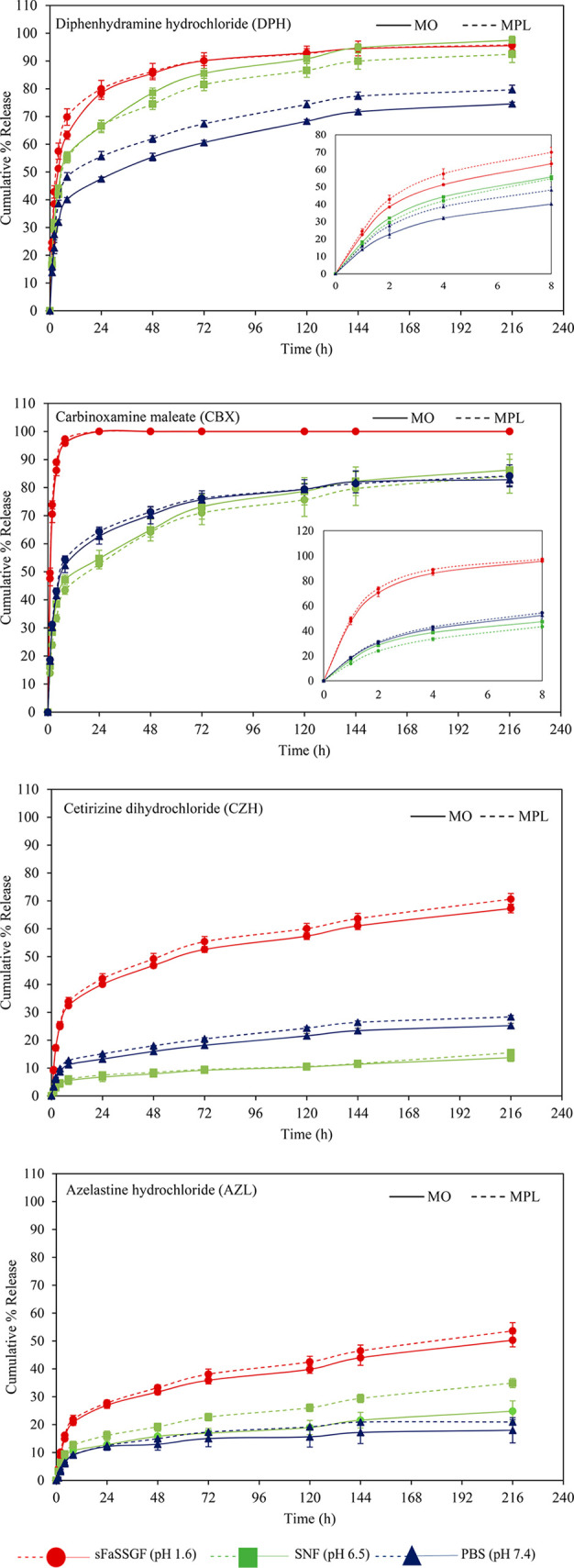
In vitro antihistamine release profiles from bulk lipid cubic formulations prepared with MO or MPL at 1 wt % drug loading incubated at 37 °C in PBS media at pH ≈ 7.4, SNF at pH 6.4, or FaSSGF at pH ≈ 1.6. Each point represents the mean (±SD) of three determinations. The lines drawn on the plots are only to aid the reader in following the progression of the dissolution.

The dissolution profiles of the antihistamines from the bulk MAG systems at 37 °C into the dissolution media are depicted in Figure 6. The dissolution profiles of the selected antihistamines demonstrated extended release profiles into the dissolution media maintained at 37 °C over a 216 h investigation period when compared to the free as-received drugs. The release followed a biphasic pattern for most of the loaded systems, with an initial burst release in the first 8 h (with the exception of CBX in FaSSGF, which was rapidly released). Although the release in this initial period was rapid, a slower release of the drug remaining in the system was observed thereafter.

The dissolution profiles from MO and MPL LCP systems exhibited comparable controlled-release properties, with a slightly prolonged antihistamine release profile seen with the MO LCP systems compared to the MPL LCP system. The variations in release, although relatively small, may be considered to be related to observed differences in the structural dimensions of the cubic phases of both systems, where differences in water channel diameter were calculated (Table 2). This results in variations in water uptake capacity, where the osmotic effect is pertinent to the ratio of the size of the incorporated molecule to the water channel diameter.^138^ It is clear from the assembled release profiles that pH was highly influential in the rate of release into the various media, as expected.

At physiological pH (7.4), the cumulative release of the two hydrophilic molecules within the first 24 h testing period was 47.5 ± 0.7 and 55.6 ± 1.8% for the DPH and 62.7 ± 2.3 and 64.3 ± 1.6% for the CBX from MO and MPL, respectively, within the first 24 h. The more hydrophobic agents (CZH and AZL) displayed much more prolonged release profiles from both systems, which was to be expected, as the release of such lipophilic molecules from the lipid cubic phase has been shown to be degradation-controlled;^59^ in the absence of lipolytic enzymes,^59,139^ the breakdown of the cubic phases is retarded. At the end of the testing period, no significant degradation of the LCP in PBS was observed, suggesting that the release of CZH and AZL from the LCP networks was limited by the breakdown of the lipid cubic network and not driven solely by simplistic diffusive mechanisms. This supports the theory that the release of the hydrophobic compounds from LCP is substantially degradation-driven.

On the basis of available literature and the solubility studies conducted here, an increase in the release rate was expected across the samples when a more acidic environment was created. SNF was chosen to represent the conditions of the nasal passage of relevance for a topical application of the antihistamine-LCP formulation. The pH of the simulated nasal fluid was slightly lower than that of the PBS buffer at pH 6.4 and followed the same pattern in the cases of the AZL and DPH, while the dissolution rate is seen to be more controlled in this pH range for CBX. A medium representing the fasted conditions in a human stomach, the so-called FaSSGF, was selected to study the release behavior of the antihistamines from the lipid formulations into media at low pH for potential oral delivery applications. For both hydrophilic antihistamines DPH and CBX, between 70 and 97% of the encapsulated drug had gone into solution within the first 8 h of testing, as shown in the magnified portions displayed on the dissolution curves, with the difference in profiles between the two different host lipid systems of different water channel diameter noted once again. Similarly in the case of the hydrophobic drugs, the release was greatly accelerated in the acidic medium, where twice the concentration of AZL was released into FaSSGF compared to PBS, and almost threefold more was released in the case of CZH over the testing period. The stability of lipid cubic gels was monitored in FaSSGF over the testing period, and a loss of 21.6 ± 5.7% of its original mass was recorded after 10 d, likely contributing to the drug release rate. Although this increase in release rate was observed for CZH at the most acidic pH, where the COOH functional group potentially remained un-ionized, both tertiary amines (Figure 1) were likely protonated and positively charged causing the jump in solution concentration; the trend deviated from that of the other molecules when considering the rate of release into SNF and PBS.

**Figure 7:**
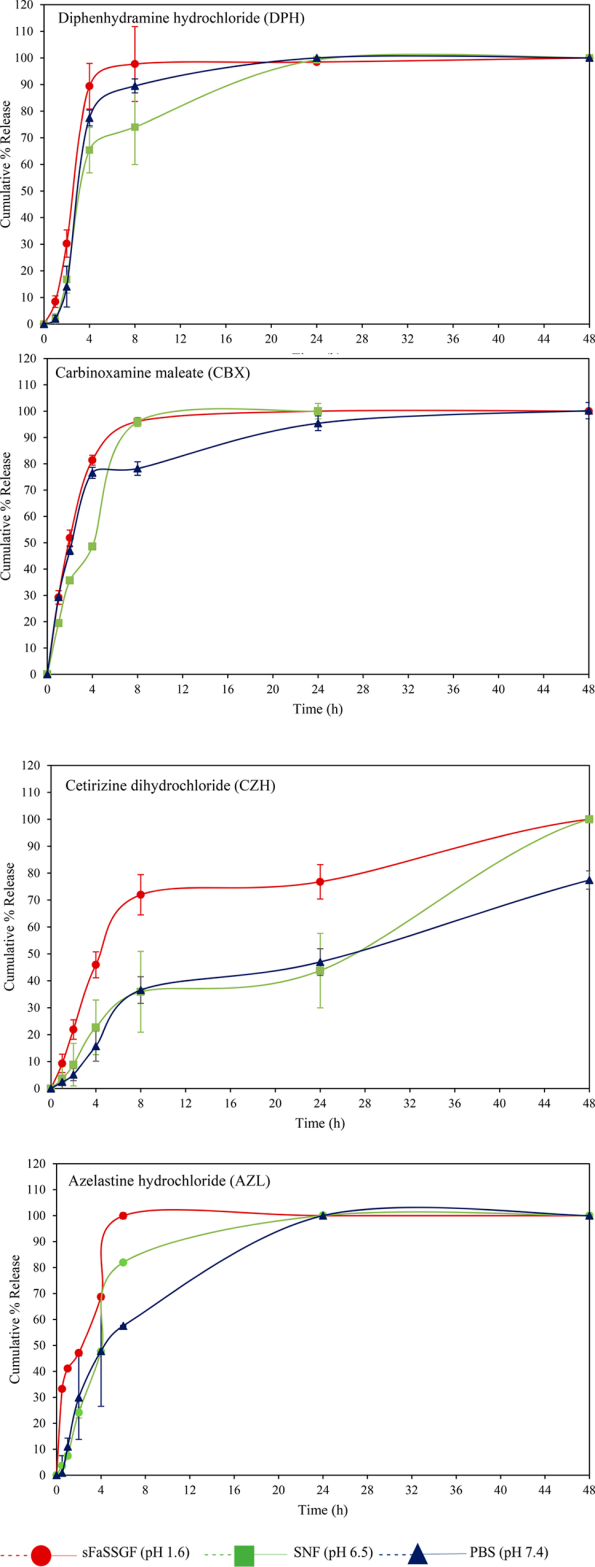
In vitro antihistamine release profiles from dispersed lipid cubic formulations (cubosomes) prepared with MO incubated at 37 °C in PBS media at pH ≈ 7.4, SNF at pH 6.4, or FaSSGF at pH ≈ 1.6. Each point represents the mean (±SD) of three determinations. The lines drawn on the plots are only to aid the reader in following the progression of the dissolution.

The duration of release from antihistamine-loaded cubosomes of MO was significantly shorter than from its bulk predecessor, where a complete release was noted within the first 48 h across all samples likely owing to the increased surface area and the dissolution of drug on or near the surface of the cubosomes. Despite the relatively low magnitude of charge on the surface of the cubosomes, Table 4, no aggregation of the cubosomes (which would be indicated as an increase in hydrodynamic radius) was observed in the dissolution media, potentially due to the steric stabilization from the added Pluronic F-127. Similar trends to those of the bulk systems were noted in the dissolution curves across all four cubosomes formulations (Figure 7), with further accelerated release observed in the case of the samples immersed in FaSSGF compared to PBS. Although the release from the cubosomes was notably faster than that observed from the bulk gel systems, the dissolution rates are significantly slower than those of the free drug in solution (Figure 5). The more lipid-soluble drugs (AZL, CZH) displayed a more prolonged release pattern compared to the other antihistamines.

Variations in the pH or ionic strength of a dissolution medium have been shown in the literature to greatly influence the release behavior of active ingredients from the lipid cubic phase causing significant changes in their profiles.^140−142^ For some drugs, the release was accelerated under acidic conditions when compared to its behavior at neutral pH.^140,141^ This has been attributed not only to differences in the solubility of the encapsulated drug within certain pH ranges as discussed above but also to changes in the lipid matrix itself. It has been suggested that the ionization of the fatty acid chain under neutral conditions may be responsible for the slowed-down release of the encapsulated drugs, whereas the acid chains are un-ionized in acidic environments.^141,143^ A “relocation” of encapsulated drugs when different pH stresses are applied has been described where the formation of less-soluble ion pairs between positively charged drugs and the negatively charged fatty acid chains of the lipids may impede their dissolution. This has been demonstrated for drugs including doxorubicin,^73^ where at neutral pH the drugs may reside in the lipid portion owed to hydrophobic tendencies, but once the pH is altered and, subsequently, the solubility and ionization state of the molecule, the drug may partition into the aqueous channels or vice versa. This will directly impact the rate of drug release, which is directly correlated with its location in the matrix.^144^ It is acknowledged that the recommended dosage requirements vary for the individual drugs tested (Supporting Information), but the lipid cubic formulations were prepared at the same loading concentration (1 wt %) to minimize the number of variables potentially affecting drug release.^145^

The pH of the cell culture media used in the cellular uptake and cytotoxicity studies, Figures 2 and 3, is comparable to that of PBS. Thus, when considering the drug release kinetics of the antihistamine-loaded cubosomes, all of the drugs would have been released into the media within the first 24 h when the cells were incubated with the cubosomes with and without the antihistamine drugs, with the exception of cetirizine dihydrochloride, (CZH). Only 50% of CZH had been released by 24 h, which could indicate a higher toxicity of the CZH or indeed a higher toxicity of CZH when loaded into the cubosomes. Of course, there may be faster/slower release in the cell culture media due to the other components present. However, if these two results, the release kinetics and the cytotoxicity, are considered with the cellular uptake results, it appears that the cubosomes may all be inside the cells within 12 h. If this is the case, carbinoxamine maleate-, azelastine hydrochloride-, diphenhydramine hydrochloride-, and cetirizine dihydrochloride-loaded cubosomes contain at minimum ∼10%, ∼20%, ∼5%, or ∼60% of the loaded drug when they get inside the cells. Given the reported much higher concentrations of cetirizine hydrochloride that showed toxicity,^113^ the cellular uptake of its cubosomes and the subsequent intracellular delivery of the drug may contribute to the observed toxicity, Figure 3.

### RBL-2H3 Inhibitory Effect Study

**Figure 8:**
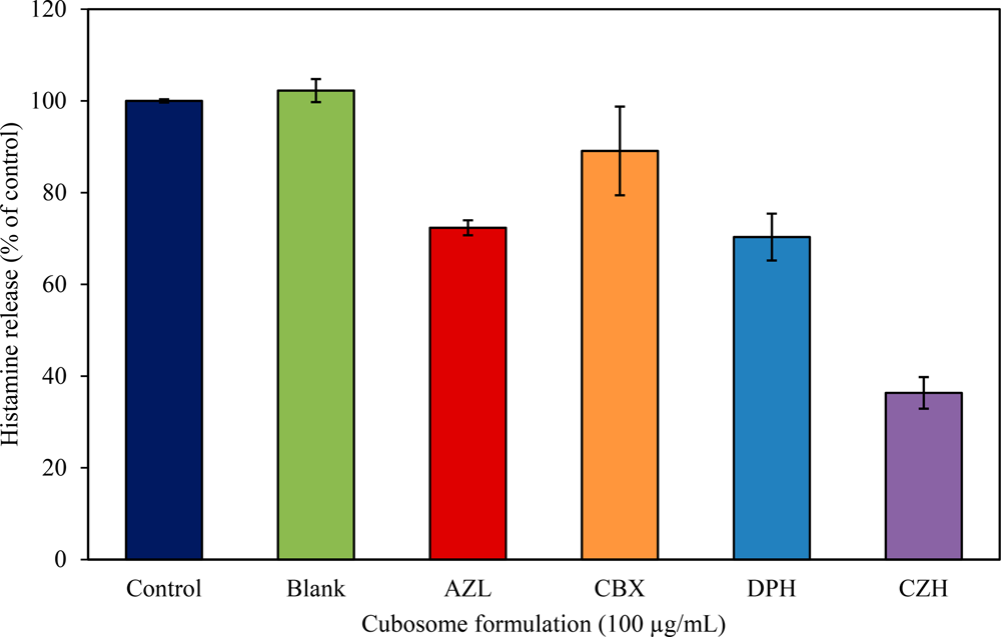
Histamine release induced in RBL-2H3 cells after an incubation for 10 min in the presence of MO cubosomes (100 μg/mL) formulated with (1 wt %) and without (Blank) antihistamine molecules. Samples were read at 450 nm and are expressed as a percentage of the control. Data are expressed as a mean ± SD of duplicated experiments.

Antihistamine molecules delivered in high concentrations have demonstrated the ability to inhibit mast cell activation and subsequent histamine release, likely through the downregulation of calcium ions in the cell, although the mechanism is still not fully understood.^9,12−14^ They have been shown to impede IgE-mediated histamine release from basophilic and mast cells in vitro.^146,147^ The RBL-2H3 cell line can be activated to secrete histamine by an aggregation of their high-affinity IgE receptors or with calcium ionophores.^148,149^ In this study, the ability of the encapsulated antihistamines (delivered at a cubosome concentration of ∼100 μg/mL corresponding to ∼1 μg/mL of drug) to inhibit a histamine release from a basophilic leukemia cell line (RBL-2H3) with known IgE-induced histamine degranulation properties was investigated. The inhibitory effect of the antihistamine formulations was quantified using a histamine ELISA kit, Figure 8. The antihistamine molecules CZH and AZL have previously shown selective H_I_-receptor antagonism and the ability to inhibit mediator release and inhibition of eosinophil migration or degranulation.^150^ Free CZH has also been shown to inhibit histamine release in RBL-2H3 cells at concentrations as low as 15 ng/mL.^65^ The inhibitory effect of first-generation antihistamine DPH against antigen-induced IgE mediated histamine release has previously been shown, where the released histamine was halved when the drug was present in concentrations up to 0.5 mM.^147^ Similarly, AZL has been found to significantly inhibit an anti-IgE-stimulated basophil histamine release,^151^ specifically in animal models.^152−154^

The cells were most sensitive to cetirizine dihydrochloride (CZH), showing that an incubation with the cubosomal formulation for as little as 10 min was sufficient in reducing the histamine release by over 60% compared to the control. The ability of CZH to inhibit a mediator release upon a pretreatment has been reported in the literature, with studies claiming that, at high dosage concentrations, the drug halted the histamine release in clinical trials in humans.^155−158^ Similar results to those seen here have been reported for a free CZH treatment at the same concentration, where a reduction in the histamine release of almost 80% from the RBL-2H3 cell model was reported.^65^ AZL and DPH loaded in cubosomes exerted a similar inhibitory effect on the histamine release, with a reduction of ∼30% observed in each case. The smallest effect was observed in the case of the hydrophilic CBX formulation, which was found to be not significantly different to that of the control (at *p* < 0.05). To our knowledge, CBX has not been reported in similar studies monitoring an IgE-stimulated histamine release. This insignificant reduction in mediator release may be explained by differences in an in vivo mediator release suppression between certain models, where the role of a histamine in an inflammatory response is dependent on the organ and complemented by the effects of other mediators such as leukotrienes and prostaglandins—which are reported to be site-specific.^11,56^

These results serve to highlight the non interfering effect of the cubosomal carrier on the effect of these molecules and, given the muco-adhesiveness and the prolonged release profiles discussed above, highlight the long-acting potential of these antihistamine cubosome formulations. An efficient accumulation of drug carriers smaller than 200 nm has previously been demonstrated in cells of certain pathologies.^91−93^ Release studies show that very little antihistamine is released from the cubosomes in 10 min, even for the fastest-releasing antihistamine, CBX, at physiological pH, Figure 7. Of course, as mentioned above, there may be a faster/slower release due to the other components of the cell culture media. Equally, the cubosomes are not fully internalized into the cells in 10 min, Figure 2. Thus, it is possible that the response seen in this study is a cumulative result of the low levels of antihistamine drug that have been released from the cubosomal dispersions before cellular uptake as well as unreleased encapsulated drug, where cells may retain or internalize the lipid nanoparticles. Longer incubation times may elicit an even greater effect. Importantly, the lipid cubic system and its associated nanodispersions, cubosomes,^89^ have been classified as potential intermediates in membrane fusion,^79,159^ where, after the adsorption of the material on the cell membrane, the two lipid bilayers fuse thus mitigating the toxicity and enhancing the cellular delivery of the targets (a process also described in the internalization of viruses^160^). These fusogenic drug carriers are capable of evading an endocytic internalization to effectively deliver targets. An important consideration in the study of drug release and uptake is the rate at which fusion occurs versus drug release, whether or not a release after an adsorption of the system to the cell surface outpaces fusion is an important consideration. It has been demonstrated here that the rate of release is highly dependent on the physiochemical properties and location of the drug molecule within the lipid system (Figures 6 and 7). It is therefore reasonable to assume that both factors may contribute to drug delivery, with one factor or the other dominating in specific circumstances and for specific lipid formulations.

## 4 Conclusions

In this work, MAG lipid cubic phases and their dispersions have shown potential as mucoadhesive controlled release systems for the delivery of four commercially available antihistamine molecules. The bioadhesive nature of these systems presents an opportunity for tapping into the improved retention, absorption, and subsequent bioavailability of the molecules through a local delivery of antihistamines. Mucosal membranes within the nasal mucosal cavity or at gastrointestinal sites could facilitate the retention of the active ingredients for the treatment of allergic reactions (this could be extended to other mucosa including buccal, vaginal,^48^ pulmonary, and those of the renal system) using these systems. Likely owing to their increased surface area, cubosomes are said to be more bioadhesive in nature than bulk gels so that they can be conveniently used in a topical and mucosal delivery of different drugs. A major challenge in the area of bioadhesive drug delivery systems is the uncontrollable hydration of bioadhesive formulations at the delivery site.^116^ The system’s resistance to dilution beyond a maximum hydration level eliminates the need for overcoming this obstacle as far as the cubosomal formulations are concerned.

Four antihistamine molecules, which currently have to be administered at minimum once a day in their current commercially available formulations, with a range of permeability and solubility properties, demonstrated prolonged release profiles from the lipid cubic phases, in both bulk and dispersed (cubosome) systems. The release pattern of the antihistamines was influenced by the drug solubility and the pH of the dissolution media. Further control of the release kinetics could be derived by an encapsulation of lipase inhibitors with the antihistamines into the lipid phase. No cytotoxic effect from the drug-loaded cubosomes was observed in two model cell lines, with the exception of CZH, which appeared to reduce the cell viability by half after a 48 h incubation in fibroblast cells. With the ultimate application in therapies for the treatment of allergic reactions, the formulations were shown to inhibit a mediator release from basophilic mast cells by more than half in some cases compared to the untreated control. This activity, combined with the prolonged release of both sets of first- and second-generation antihistamine molecules from the bulk and dispersed systems compared to the free drugs, highlights the potential of these systems as easy-to-apply long-acting antihistamine medications.

## Supporting Information Available

Properties, formulations, and indications of the antihistamines investigated in this study, calibration curves for first- and second-generation antihistamines obtained by HPLC, additional MP-SPR sensograms and the kinetics of adsorption to mucin as measured by MP-SPR, diffractograms of the as-received antihistamine molecules and 1D azimuthally integrated SAXS patterns of (i) bulk MO and MPL cubic mesophases formulated with or without antihistamines and (ii) MO cubosomes formulated with antihistamines (PDF)

## References

[ref1] Best C. H.; et al. The nature of the vaso-dilator constituents of certain tissue extracts. J. Physiol. 1927, 62 (4), 397–417. 10.1113/jphysiol.1927.sp002369. 16993860 PMC1514980

[ref2] Loew E. R.; Chickering O. Gastric secretion in dogs treated with histamine antagonist, thymoxyethyldiethylamine. Exp. Biol. Med. 1941, 48 (1), 65–68. 10.3181/00379727-48-13221.

[ref3] Black J.; et al. Definition and antagonism of histamine H2-receptors. Nature 1972, 236, 385–390. 10.1038/236385a0. 4401751

[ref4] Wollenberg A.; Feichtner K. Atopic dermatitis and skin allergies–update and outlook. Allergy 2013, 68 (12), 1509–1519. 10.1111/all.12324. 24410780

[ref5] Hide M.; et al. Autoantibodies against the high-affinity IgE receptor as a cause of histamine release in chronic urticaria. N. Engl. J. Med. 1993, 328 (22), 1599–1604. 10.1056/NEJM199306033282204. 7683772

[ref6] Walch H. Topical application of cetirizine and loratadine. U.S. Patent US6790847B2, 2004.

[ref7] Heinrich J.; et al. European health survey in adults (ECRHS). Pneumologie 2002, 56 (5), 297–303. 10.1055/s-2002-30699. 12089647

[ref8] World Allergy Organization (WAO) White book on allergy: update 2013*;* WAO: Milwaukee, WI, 2013.

[ref9] Simons F. E. R.; Simons K. J. Histamine and H1-antihistamines: celebrating a century of progress. J. Allergy Clin. Immunol. 2011, 128 (6), 1139–1150. 10.1016/j.jaci.2011.09.005. 22035879

[ref10] Leurs R.; Church M.; Taglialatela M. H1-antihistamines: inverse agonism, anti-inflammatory actions and cardiac effects. Clin. Exp. Allergy 2002, 32 (4), 489–498. 10.1046/j.0954-7894.2002.01314.x. 11972592

[ref11] Simons F. E. R. The antiallergic effects of antihistamines (H1-receptor antagonists). J. Allergy Clin. Immunol. 1992, 90 (4), 705–715. 10.1016/0091-6749(92)90156-V. 1383310

[ref12] Simons F. E. R. Advances in H1-antihistamines. N. Engl. J. Med. 2004, 351 (21), 2203–2217. 10.1056/NEJMra033121. 15548781

[ref13] Bakker R. A.; et al. Histamine H1-receptor activation of nuclear factor-κB: roles for Gβγ-and Gαq/11-subunits in constitutive and agonist-mediated signaling. Mol. Pharmacol. 2001, 60 (5), 1133–1142. 10.1124/mol.60.5.1133. 11641442

[ref14] Weller K.; Maurer M. Desloratadine inhibits human skin mast cell activation and histamine release. J. Invest. Dermatol. 2009, 129 (11), 2723 10.1038/jid.2009.134. 19516262

[ref15] Chishty M.; et al. Affinity for the P-glycoprotein efflux pump at the blood-brain barrier may explain the lack of CNS side-effects of modern antihistamines. J. Drug Targeting 2001, 9 (3), 223–228. 10.3109/10611860108997930. 11697207

[ref16] Timmerman H. Factors involved in the absence of sedative effects by the second-generation antihistamines. Allergy 2000, 55, 5–10. 10.1034/j.1398-9995.2000.055supp60005.x. 10887969

[ref17] Chen C.; et al. P-glycoprotein limits the brain penetration of nonsedating but not sedating H1-antagonists. Drug Metab. Dispos. 2003, 31 (3), 312–318. 10.1124/dmd.31.3.312. 12584158

[ref18] Machado O. L. T.; de Campos-Mesquita D. M.; Pacheco-Soares T. Antihistaminic Treatment, Allergen-Specific Immunotherapy, and Blockade of IgE as Alternative Allergy Treatments. Allergen 2017, 67 10.5772/intechopen.69912.

[ref19] Church M. K.; Church D. S. Pharmacology of antihistamines. Indian journal of dermatology 2013, 58 (3), 219 10.4103/0019-5154.110832. 23723474 PMC3667286

[ref20] Wang C.; Hu S.; Sun C. C. Expedited development of diphenhydramine orally disintegrating tablet through integrated crystal and particle engineering. Mol. Pharmaceutics 2017, 14 (10), 3399–3408. 10.1021/acs.molpharmaceut.7b00423. 28825961

[ref21] Morita Y.; Koyama N.; Ohsawa S. U.S. Patent US6117864A, Methods employing stable preparation containing azelastine hydrochloride. 2000.

[ref22] Van Eeckhaut A.; et al. Influence of methanol on the enantioresolution of antihistamines with carboxymethyl-β-cyclodextrin in capillary electrophoresis. Electrophoresis 2004, 25 (16), 2838–2847. 10.1002/elps.200406021. 15352017

[ref23] Bajerski L.; et al. Determination of cetirizine in tablets and compounded capsules: comparative study between CE and HPLC. Quim. Nova 2010, 33 (1), 114–118. 10.1590/S0100-40422010000100021.

[ref24] Shin S.-C.; Yoon M.-K. Application of TPX polymer membranes for the controlled release of triprolidine. Int. J. Pharm. 2002, 232 (1–2), 131–137. 10.1016/S0378-5173(01)00906-1. 11790496

[ref25] Hindmarch I.; Shamsi Z. Antihistamines: models to assess sedative properties, assessment of sedation, safety and other side-effects. Clin. Exp. Allergy 1999, 29, 133–142. 10.1046/j.1365-2222.1999.0290s3133.x. 10444227

[ref26] Jain G. K.; Rampal A.; Sen H. Process for the preparation of a controlled drug delivery system containing pseudoephedrine and a long acting antihistamine. WIPO Patent WO2001021168A1, 2001.

[ref27] Rabinowitz J. D.; Zaffaroni A.C. Delivery of antihistamines through an inhalation route. U.S. Patent US20040156789A1, 2004.

[ref28] Gu X.; et al. Evaluation and comparison of five matrix excipients for the controlled release of acrivastine and pseudoephedrine. Drug Dev. Ind. Pharm. 2004, 30 (10), 1009–1017. 10.1081/DDC-200040237. 15595567

[ref29] Goindi S.; Dhatt B.; Kaur A. Ethosomes-based topical delivery system of antihistaminic drug for treatment of skin allergies. J. Microencapsulation 2014, 31 (7), 716–724. 10.3109/02652048.2014.918667. 24963956

[ref30] Rossi A.; et al. A preliminary study on topical cetirizine in the therapeutic management of androgenetic alopecia. J. Dermatol. Treat. 2018, 29 (2), 149–151. 10.1080/09546634.2017.1341610. 28604133

[ref31] ElzainyA. A.; et al. Cetirizine from topical phosphatidylcholine-hydrogenated liposomes: evaluation of peripheral antihistaminic activity and systemic absorption in a rabbit model. AAPS J. 2004, 6 (3), 7–12. 10.1208/aapsj060318.15760103

[ref32] Shin S.-C.; Lee H.-J. Controlled release of triprolidine using ethylene-vinyl acetate membrane and matrix systems. Eur. J. Pharm. Biopharm. 2002, 54 (2), 201–206. 10.1016/S0939-6411(02)00051-6. 12191692

[ref33] Ramachandran S.; Nandhakumar S.; Dhanaraju M. D. Formulation and characterization of glutaraldehyde cross-linked chitosan biodegradable microspheres loaded with famotidine. Trop. J. Pharm. Res., 2011. 10( (3), ). 10.4314/tjpr.v10i3.13

[ref34] Bize C.; et al. Bioactive Formulations with Sugar-Derived Surfactants: A New Approach for Photoprotection and Controlled Release of Promethazine. ChemPhysChem 2013, 14 (6), 1126–1131. 10.1002/cphc.201200932. 23436492

[ref35] Rizwan S.; et al. Characterisation of bicontinuous cubic liquid crystalline systems of phytantriol and water using cryo field emission scanning electron microscopy (cryo FESEM). Micron 2007, 38 (5), 478–485. 10.1016/j.micron.2006.08.003. 17011783

[ref36] Kumar R.; Philip A. Modified transdermal technologies: Breaking the barriers of drug permeation via the skin. Trop. J. Pharm. Res. 2007, 6 (1), 633–644. 10.4314/tjpr.v6i1.14641.

[ref37] Chien Y. W. Logics of transdermal controlled drug administration. Drug Dev. Ind. Pharm. 1983, 9 (4), 497–520. 10.3109/03639048309044691.

[ref38] Cevc G. Drug delivery across the skin. Expert Opin. Invest. Drugs 1997, 6 (12), 1887–1937. 10.1517/13543784.6.12.1887. 15989590

[ref39] Kalepu S.; Manthina M.; Padavala V. Oral lipid-based drug delivery systems–an overview. Acta Pharm. Sin. B 2013, 3 (6), 361–372. 10.1016/j.apsb.2013.10.001.

[ref40] Shrestha H.; Bala R.; Arora S. Lipid-based drug delivery systems. J. Pharm. 2014, 2014, 1 10.1155/2014/801820. PMC4590796 26556202

[ref41] Clogston J. Applications of the lepidic cubic phase: from controlled release and uptake to in meso crystallization of membrane proteins*;* The Ohio State University, 2005.

[ref42] Qiu H.; Caffrey M. The phase diagram of the monoolein/water system: metastability and equilibrium aspects. Biomaterials 2000, 21 (3), 223–234. 10.1016/S0142-9612(99)00126-X. 10646938

[ref43] Tanford C. The hydrophobic effect and the organization of living matter. Science 1978, 200 (4345), 1012–1018. 10.1126/science.653353. 653353

[ref44] Nielsen L. S.; Schubert L.; Hansen J. Bioadhesive drug delivery systems: I. Characterisation of mucoadhesive properties of systems based on glyceryl mono-oleate and glyceryl monolinoleate. Eur. J. Pharm. Sci. 1998, 6 (3), 231–239. 10.1016/S0928-0987(97)10004-5. 9795071

[ref45] Hundekar Y. R.; et al. Preparation and evaluation of diclofenac sodium cubosomes for percutaneous administration. World journal of pharmacy and pharmaceutical sciences 2014, 3 (1), 523–539.

[ref46] Kulkarni C. V.; et al. Monoolein: a magic lipid?. Phys. Chem. Chem. Phys. 2011, 13 (8), 3004–3021. 10.1039/C0CP01539C. 21183976

[ref47] Müller R., Souto E.; Radtke M. Medicament vehicle for the controlled administration of an active agent, produced from lipid matrix-medicament conjugates. WIPO Patent WO2000067800A3, 2000.

[ref48] Shah J. C.; Sadhale Y.; Chilukuri D. M. Cubic phase gels as drug delivery systems. Adv. Drug Delivery Rev. 2001, 47 (2–3), 229–250. 10.1016/S0169-409X(01)00108-9. 11311994

[ref49] Siekmann B.; et al. Preparation and structural investigations of colloidal dispersions prepared from cubic monoglyceride–water phases. Int. J. Pharm. 2002, 244 (1–2), 33–43. 10.1016/S0378-5173(02)00298-3. 12204563

[ref50] Attama A. A.; Müller-Goymann C. C. Effect of beeswax modification on the lipid matrix and solid lipid nanoparticle crystallinity. Colloids Surf., A 2008, 315 (1–3), 189–195. 10.1016/j.colsurfa.2007.07.035.

[ref51] Forbes R.; Cooper A.; Mitchell H. The composition of the adult human body as determined by chemical analysis. J. Biol. Chem. 1953, 203 (1), 359–366. 10.1016/S0021-9258(19)52646-1. 13069519

[ref52] Engström S.; et al. A study of polar lipid drug systems undergoing a thermoreversible lamellar-to-cubic phase transition. Int. J. Pharm. 1992, 86 (2–3), 137–145. 10.1016/0378-5173(92)90190-D.

[ref53] Bender J.; et al. Lipid cubic phases for improved topical drug delivery in photodynamic therapy. J. Controlled Release 2005, 106 (3), 350–360. 10.1016/j.jconrel.2005.05.010. 15967535

[ref54] Chang C.-M.; Bodmeier R. Monoglyceride based liquid crystalline topical drug delivery systems. Pharm. Res. 1994, 11, S185.

[ref55] Gan L.; et al. Recent advances in topical ophthalmic drug delivery with lipid-based nanocarriers. Drug Discovery Today 2013, 18 (5–6), 290–297. 10.1016/j.drudis.2012.10.005. 23092895

[ref56] Du Buske L. M. Clinical comparison of histamine H1–receptor antagonist drugs. J. Allergy Clin. Immunol. 1996, 98 (6), S307–S318. 10.1016/S0091-6749(96)80116-3. 8977542

[ref57] Wu H.; et al. A novel small Odorranalectin-bearing cubosomes: Preparation, brain delivery and pharmacodynamic study on amyloid-β25–35-treated rats following intranasal administration. Eur. J. Pharm. Biopharm. 2012, 80 (2), 368–378. 10.1016/j.ejpb.2011.10.012. 22061263

[ref58] Boge L. Lipid-based liquid crystals as drug delivery vehicles for antimicrobial peptides*;* Chalmers Tekniska Hogskola: Sweden, 2018

[ref59] Dully M.; et al. Modulating the Release of Pharmaceuticals from Lipid Cubic Phases using a Lipase Inhibitor. J. Colloid Interface Sci. 2020, 573 (573), 176–192. 10.1016/j.jcis.2020.04.015. 32278949

[ref60] Azhari H.; et al. Stabilising cubosomes with Tween 80 as a step towards targeting lipid nanocarriers to the blood–brain barrier. Eur. J. Pharm. Biopharm. 2016, 104, 148–155. 10.1016/j.ejpb.2016.05.001. 27163239

[ref61] Anderson D. M.; Gruner S. M.; Leibler S. Geometrical aspects of the frustration in the cubic phases of lyotropic liquid crystals. Proc. Natl. Acad. Sci. U. S. A. 1988, 85 (15), 5364–5368. 10.1073/pnas.85.15.5364. 3399497 PMC281757

[ref62] Turner D. C.; et al. Structural study of the inverted cubic phases of di-dodecyl alkyl-β-D-glucopyranosyl-rac-glycerol. J. Phys. II 1992, 2 (11), 2039–2063. 10.1051/jp2:1992250.

[ref63] Prosperi-Porta G.; et al. Phenylboronic-acid-based polymeric micelles for mucoadhesive anterior segment ocular drug delivery. Biomacromolecules 2016, 17 (4), 1449–1457. 10.1021/acs.biomac.6b00054. 26963738

[ref64] Farid R. M.; et al. Formulation and in vitro evaluation of salbutamol sulphate in situ gelling nasal inserts. AAPS PharmSciTech 2013, 14 (2), 712–718. 10.1208/s12249-013-9956-y. 23516112 PMC3666017

[ref65] Hussein Al Ali S.; Hussein Al Ali S.; Hussein M. Z.; Zainal Z.; Nazrul-Hakim M.; Al-Qubaisi M.; Ismail M.; et al. Controlled-release formulation of antihistamine based on cetirizine zinc-layered hydroxide nanocomposites and its effect on histamine release from basophilic leukemia (RBL-2H3) cells. Int. J. Nanomed. 2012, 7, 3351 10.2147/IJN.S30809. PMC3405893 22848164

[ref66] Chung H.; Caffrey M. Polymorphism, mesomorphism, and metastability of monoelaidin in excess water. Biophys. J. 1995, 69 (5), 1951–1963. 10.1016/S0006-3495(95)80065-2. 8580338 PMC1236428

[ref67] Briggs J. The phase behavior of hydrated monoacylglycerols and the design of an X-ray compatible scanning calorimeter*;* The Ohio State University, 1994.

[ref68] Eriksson P.-O.; Lindblom G. Lipid and water diffusion in bicontinuous cubic phases measured by NMR. Biophys. J. 1993, 64 (1), 129–136. 10.1016/S0006-3495(93)81347-X. 8431537 PMC1262309

[ref69] Norling T.; et al. Formulation of a drug delivery system based on a mixture of monoglycerides and triglycerides for use in the treatment of periodontal disease. J. Clin. Periodontol. 1992, 19 (9), 687–692. 10.1111/j.1600-051X.1992.tb02529.x. 1447387

[ref70] Barauskas J.; Landh T. Phase behavior of the phytantriol/water system. Langmuir 2003, 19 (23), 9562–9565. 10.1021/la0350812.

[ref71] Hato M.; et al. Phase behavior of phytanyl-chained akylglycoside/water systems. Trends in Colloid and Interface Science XVI 2004, 56–60. 10.1007/978-3-540-36462-7_14.

[ref72] Briggs J.; Chung H.; Caffrey M. The temperature-composition phase diagram and mesophase structure characterization of the monoolein/water system. J. Phys. II 1996, 6 (5), 723–751. 10.1051/jp2:1996208.

[ref73] Nazaruk E.; et al. Design and assembly of pH-sensitive lipidic cubic phase matrices for drug release. Langmuir 2014, 30 (5), 1383–1390. 10.1021/la403694e. 24443890

[ref74] Caboi F.; et al. Structural effects, mobility, and redox behavior of vitamin K1 hosted in the monoolein/water liquid crystalline phases. Langmuir 1997, 13 (20), 5476–5483. 10.1021/la9702188.

[ref75] Yaghmur A. Tuning curvature and stability of monoolein bilayers by designer lipid-like peptide surfactants. PLoS One 2007, 2 (5), e479 10.1371/journal.pone.0000479. 17534429 PMC1868779

[ref76] Borné J.; Nylander T.; Khan A. Phase behavior and aggregate formation for the aqueous monoolein system mixed with sodium oleate and oleic acid. Langmuir 2001, 17 (25), 7742–7751. 10.1021/la010650w.

[ref77] Angelov B.; et al. Long-living intermediates during a lamellar to a diamond-cubic lipid phase transition: a small-angle X-ray scattering investigation. Langmuir 2009, 25 (6), 3734–3742. 10.1021/la804225j. 19708151

[ref78] Nakano M.; et al. Small-angle X-ray scattering and 13C NMR investigation on the internal structure of “cubosomes. Langmuir 2001, 17 (13), 3917–3922. 10.1021/la010224a.

[ref79] Larsson K. Cubic lipid-water phases: structures and biomembrane aspects. J. Phys. Chem. 1989, 93 (21), 7304–7314. 10.1021/j100358a010.

[ref80] Gustafsson J.; et al. Cubic lipid– water phase dispersed into submicron particles. Langmuir 1996, 12 (20), 4611–4613. 10.1021/la960318y.

[ref81] Gustafsson J.; et al. Submicron particles of reversed lipid phases in water stabilized by a nonionic amphiphilic polymer. Langmuir 1997, 13 (26), 6964–6971. 10.1021/la970566+.

[ref82] Yaghmur A. Self-assembly in monoelaidin aqueous dispersions: direct vesicles to cubosomes transition. PLoS One 2008, 3 (11), e3747 10.1371/journal.pone.0003747. 19015726 PMC2581612

[ref83] Nakano M.; et al. Dispersions of liquid crystalline phases of the monoolein/oleic acid/Pluronic F127 system. Langmuir 2002, 18 (24), 9283–9288. 10.1021/la026297r.

[ref84] Mozafari M. Nanoliposomes: preparation and analysis. In Liposomes*;* Springer, 2010; pp 29–50. 10.1007/978-1-60327-360-2_2 20072871

[ref85] Sou K. Electrostatics of carboxylated anionic vesicles for improving entrapment capacity. Chem. Phys. Lipids 2011, 164 (3), 211–215. 10.1016/j.chemphyslip.2011.01.002. 21262210

[ref86] Han S.; et al. Novel vehicle based on cubosomes for ophthalmic delivery of flurbiprofen with low irritancy and high bioavailability. Acta Pharmacol. Sin. 2010, 31 (8), 990–998. 10.1038/aps.2010.98. 20686524 PMC4007820

[ref87] Levy M.; et al. Characterization of diazepam submicron emulsion interface: role of oleic acid. J. Microencapsulation 1994, 11 (1), 79–92. 10.3109/02652049409040440. 8138877

[ref88] Freitas C.; Müller R. H. Effect of light and temperature on zeta potential and physical stability in solid lipid nanoparticle (SLN) dispersions. Int. J. Pharm. 1998, 168 (2), 221–229. 10.1016/S0378-5173(98)00092-1.

[ref89] Barriga H. M.; Holme M. N.; Stevens M. M. Cubosomes: the next generation of smart lipid nanoparticles?. Angew. Chem., Int. Ed. 2019, 58 (10), 2958–2978. 10.1002/anie.201804067. PMC6606436 29926520

[ref90] Tilley A. J.; Drummond C. J.; Boyd B. J. Disposition and association of the steric stabilizer Pluronic® F127 in lyotropic liquid crystalline nanostructured particle dispersions. J. Colloid Interface Sci. 2013, 392, 288–296. 10.1016/j.jcis.2012.09.051. 23137909

[ref91] Diepold R.; et al. Distribution of poly-hexyl-2-cyano-[3–14C] acrylate nanoparticles in healthy and chronically inflamed rabbit eyes. Int. J. Pharm. 1989, 54 (2), 149–153. 10.1016/0378-5173(89)90334-7.

[ref92] Duncan R.; Connors T.A.; Meada H. Drug targeting in cancer therapy: the magic bullet, what next? Taylor & Francis, 1996. 10.3109/10611869608996823 8866651

[ref93] Monsky W. L.; et al. Augmentation of transvascular transport of macromolecules and nanoparticles in tumors using vascular endothelial growth factor. Cancer Res. 1999, 59 (16), 4129–4135. 10463618

[ref94] Guo C.; et al. Lyotropic liquid crystal systems in drug delivery. Drug Discovery Today 2010, 15 (23–24), 1032–1040. 10.1016/j.drudis.2010.09.006. 20934534

[ref95] Nafee N.; et al. Relevance of the colloidal stability of chitosan/PLGA nanoparticles on their cytotoxicity profile. Int. J. Pharm. 2009, 381 (2), 130–139. 10.1016/j.ijpharm.2009.04.049. 19450671

[ref96] Chung T.-H.; et al. The effect of surface charge on the uptake and biological function of mesoporous silica nanoparticles in 3T3-L1 cells and human mesenchymal stem cells. Biomaterials 2007, 28 (19), 2959–2966. 10.1016/j.biomaterials.2007.03.006. 17397919

[ref97] Zhang D.; et al. The morphology and surface charge-dependent cellular uptake efficiency of upconversion nanostructures revealed by single-particle optical microscopy. Chemical science 2018, 9 (23), 5260–5269. 10.1039/C8SC01828F. 29997881 PMC6001388

[ref98] Selvi R. B.; et al. ATP driven clathrin dependent entry of carbon nanospheres prefer cells with glucose receptors. J. Nanobiotechnol. 2012, 10 (1), 35 10.1186/1477-3155-10-35. PMC3479219 22857258

[ref99] Wilhelm C.; et al. Intracellular uptake of anionic superparamagnetic nanoparticles as a function of their surface coating. Biomaterials 2003, 24 (6), 1001–1011. 10.1016/S0142-9612(02)00440-4. 12504522

[ref100] Lorenz M. R.; et al. Uptake of functionalized, fluorescent-labeled polymeric particles in different cell lines and stem cells. Biomaterials 2006, 27 (14), 2820–2828. 10.1016/j.biomaterials.2005.12.022. 16430958

[ref101] Sahay G.; Alakhova D. Y.; Kabanov A. V. Endocytosis of nanomedicines. J. Controlled Release 2010, 145 (3), 182–195. 10.1016/j.jconrel.2010.01.036. PMC2902597 20226220

[ref102] Martins S.; et al. Solid lipid nanoparticles as intracellular drug transporters: an investigation of the uptake mechanism and pathway. Int. J. Pharm. 2012, 430 (1–2), 216–227. 10.1016/j.ijpharm.2012.03.032. 22465548

[ref103] Ravi P. R.; et al. Lipid nanoparticles for oral delivery of raloxifene: optimization, stability, in vivo evaluation and uptake mechanism. Eur. J. Pharm. Biopharm. 2014, 87 (1), 114–124. 10.1016/j.ejpb.2013.12.015. 24378615

[ref104] Kam N. W. S.; Liu Z.; Dai H. Carbon nanotubes as intracellular transporters for proteins and DNA: an investigation of the uptake mechanism and pathway. Angew. Chem., Int. Ed. 2006, 45 (4), 577–581. 10.1002/anie.200503389. 16345107

[ref105] Thurn K. T.; et al. Endocytosis of titanium dioxide nanoparticles in prostate cancer PC-3M cells. Nanomedicine 2011, 7 (2), 123–130. 10.1016/j.nano.2010.09.004. 20887814 PMC3062699

[ref106] Yang Z.; et al. Evaluating the potential of cubosomal nanoparticles for oral delivery of amphotericin B in treating fungal infection. Int. J. Nanomed. 2014, 9, 327 10.2147/IJN.S54967. PMC3888350 24421641

[ref107] Prange J. A.; et al. Overcoming endocytosis deficiency by cubosome nanocarriers. ACS Applied Bio Materials 2019, 2 (6), 2490–2499. 10.1021/acsabm.9b00187. 35030705

[ref108] Luo Q.; et al. A novel glyceryl monoolein-bearing cubosomes for gambogenic acid: preparation, cytotoxicity and intracellular uptake. Int. J. Pharm. 2015, 493 (1–2), 30–39. 10.1016/j.ijpharm.2015.07.036. 26209071

[ref109] Fröhlich E. The role of surface charge in cellular uptake and cytotoxicity of medical nanoparticles. Int. J. Nanomed. 2012, 7, 5577 10.2147/IJN.S36111. PMC3493258 23144561

[ref110] Pelle E.; et al. Identification of histamine receptors and reduction of squalene levels by an antihistamine in sebocytes. J. Invest. Dermatol. 2008, 128 (5), 1280–1285. 10.1038/sj.jid.5701160. 18007585

[ref111] Snyder R. D.; Green J. W. A review of the genotoxicity of marketed pharmaceuticals. Mutat. Res., Rev. Mutat. Res. 2001, 488 (2), 151–169. 10.1016/S1383-5742(01)00055-2. 11344042

[ref112] Ellegaard A.-M.; et al. Repurposing cationic amphiphilic antihistamines for cancer treatment. EBioMedicine 2016, 9, 130–139. 10.1016/j.ebiom.2016.06.013. 27333030 PMC4972561

[ref113] Salimi A.; Razian M.; Pourahmad J. Analysis of toxicity effects of buspirone, cetirizine and olanzapine on human blood lymphocytes: in vitro model. Curr. Clin. Pharmacol. 2018, 13 (2), 120–127. 10.2174/1574884713666180516112920. 29766823

[ref114] Chung H.; et al. Self-assembled “nanocubicle” as a carrier for peroral insulin delivery. Diabetologia 2002, 45 (3), 448–451. 10.1007/s00125-001-0751-z. 11914752

[ref115] Rosa A.; et al. Monoolein-based cubosomes affect lipid profile in HeLa cells. Chem. Phys. Lipids 2015, 191, 96–105. 10.1016/j.chemphyslip.2015.08.017. 26341749

[ref116] Eldem T.; Speiser P. Intestinal fat absorption and its relevance in lipid drug delivery systems. Pharmazie 1989, 44 (7), 444–447. 2682676

[ref117] Illum L. Nasal drug delivery—possibilities, problems and solutions. J. Controlled Release 2003, 87 (1–3), 187–198. 10.1016/S0168-3659(02)00363-2. 12618035

[ref118] Mura P.; et al. In situ mucoadhesive-thermosensitive liposomal gel as a novel vehicle for nasal extended delivery of opiorphin. Eur. J. Pharm. Biopharm. 2018, 122, 54–61. 10.1016/j.ejpb.2017.10.008. 29032194

[ref119] Campbell C.; et al. Drug development of intranasally delivered peptides. Ther. Delivery 2012, 3 (4), 557–568. 10.4155/tde.12.12. 22834082

[ref120] Ghori M. U.; et al. Nasal drug delivery systems: an overview. American Journal of Pharmacological Sciences 2015, 3 (5), 110–119. 10.12691/ajps-3-5-2.

[ref121] Lochhead J. J.; Thorne R. G. Intranasal delivery of biologics to the central nervous system. Adv. Drug Delivery Rev. 2012, 64 (7), 614–628. 10.1016/j.addr.2011.11.002. 22119441

[ref122] Kim Y. S.; Ho S. B. Intestinal goblet cells and mucins in health and disease: recent insights and progress. Current gastroenterology reports 2010, 12 (5), 319–330. 10.1007/s11894-010-0131-2. 20703838 PMC2933006

[ref123] Bøgh M.; et al. Mucosal drug delivery: barriers, in vitro models and formulation strategies. J. Drug Delivery Sci. Technol. 2013, 23 (4), 383–391. 10.1016/S1773-2247(13)50055-4.

[ref124] Bansil R.; Stanley E.; LaMont J. T. Mucin biophysics. Annu. Rev. Physiol. 1995, 57 (1), 635–657. 10.1146/annurev.ph.57.030195.003223. 7778881

[ref125] Thornton D. J.; Sheehan J. K. From mucins to mucus: toward a more coherent understanding of this essential barrier. Proc. Am. Thorac. Soc. 2004, 1 (1), 54–61. 10.1513/pats.2306016. 16113413

[ref126] Carvalho F. C.; et al. Mucoadhesive drug delivery systems. Braz. J. Pharm. Sci. 2010, 46 (1), 1–17. 10.1590/S1984-82502010000100002.

[ref127] Li L. D.; et al. Spatial configuration and composition of charge modulates transport into a mucin hydrogel barrier. Biophys. J. 2013, 105 (6), 1357–1365. 10.1016/j.bpj.2013.07.050. 24047986 PMC3785869

[ref128] Bravo-Osuna I.; et al. Interfacial interaction between transmembrane ocular mucins and adhesive polymers and dendrimers analyzed by surface plasmon resonance. Pharm. Res. 2012, 29 (8), 2329–2340. 10.1007/s11095-012-0761-1. 22565639 PMC3867740

[ref129] Joergensen L.; et al. New insights into the mucoadhesion of pectins by AFM roughness parameters in combination with SPR. Int. J. Pharm. 2011, 411 (1–2), 162–168. 10.1016/j.ijpharm.2011.04.001. 21501673

[ref130] Zeng W.; et al. Hyaluronic acid-coated niosomes facilitate tacrolimus ocular delivery: Mucoadhesion, precorneal retention, aqueous humor pharmacokinetics, and transcorneal permeability. Colloids Surf., B 2016, 141, 28–35. 10.1016/j.colsurfb.2016.01.014. 26820107

[ref131] Marttin E.; et al. Nasal mucociliary clearance as a factor in nasal drug delivery. Adv. Drug Delivery Rev. 1998, 29 (1–2), 13–38. 10.1016/S0169-409X(97)00059-8. 10837578

[ref132] Ugwoke M. I.; et al. Nasal mucoadhesive drug delivery: background, applications, trends and future perspectives. Adv. Drug Delivery Rev. 2005, 57 (11), 1640–1665. 10.1016/j.addr.2005.07.009. 16182408

[ref133] Jiang L.; et al. The application of mucoadhesive polymers in nasal drug delivery. Drug Dev. Ind. Pharm. 2010, 36 (3), 323–336. 10.3109/03639040903170750. 19735210

[ref134] Wang C.; et al. Relationships among crystal structures, mechanical properties, and tableting performance probed using four salts of diphenhydramine. Cryst. Growth Des. 2017, 17 (11), 6030–6040. 10.1021/acs.cgd.7b01153.

[ref135] Reddy M.; Polymorphic forms of dihydrochloride salts of cetirizine and processes for preparation thereof. U.S. Patent US20040186112A1, 2004.

[ref136] Maccaroni E.; et al. Azelastine hydrochloride: A powder diffraction and 13C CPMAS NMR study of its anhydrous and solvated forms. Cryst. Growth Des. 2009, 9 (1), 517–524. 10.1021/cg800773v.

[ref137] Deng Y.; et al. Studies on the in vitro ion exchange kinetics and thermodynamics and in vivo pharmacokinetics of the carbinoxamine-resin complex. Int. J. Pharm. 2020, 588, 119779 10.1016/j.ijpharm.2020.119779. 32805380

[ref138] Clogston J.; Caffrey M. Controlling release from the lipidic cubic phase. Amino acids, peptides, proteins and nucleic acids. J. Controlled Release 2005, 107 (1), 97–111. 10.1016/j.jconrel.2005.05.015. 15990192

[ref139] Borné J.; Nylander T.; Khan A. Effect of lipase on different lipid liquid crystalline phases formed by oleic acid based acylglycerols in aqueous systems. Langmuir 2002, 18 (23), 8972–8981. 10.1021/la020377d.

[ref140] Negrini R.; Mezzenga R. pH-responsive lyotropic liquid crystals for controlled drug delivery. Langmuir 2011, 27 (9), 5296–5303. 10.1021/la200591u. 21452814

[ref141] Chang C.-M.; Bodmeier R. Effect of dissolution media and additives on the drug release from cubic phase delivery systems. J. Controlled Release 1997, 46 (3), 215–222. 10.1016/S0168-3659(96)01596-9.

[ref142] Rahanyan-Kägi N.; et al. Stimuli-Responsive Lipidic Cubic Phase: Triggered Release and Sequestration of Guest Molecules. Chem. - Eur. J. 2015, 21 (5), 1873–1877. 10.1002/chem.201405580. 25512248

[ref143] Vervaeck A.; et al. Prilling of fatty acids as a continuous process for the development of controlled release multiparticulate dosage forms. Eur. J. Pharm. Biopharm. 2013, 85 (3), 587–596. 10.1016/j.ejpb.2013.02.003. 23474381

[ref144] Clogston J.; et al. Controlling release from the lipidic cubic phase by selective alkylation. J. Controlled Release 2005, 102 (2), 441–461. 10.1016/j.jconrel.2004.10.007. 15653163

[ref145] Burrows R.; Collett J.; Attwood D. The release of drugs from monoglyceride-water liquid crystalline phases. Int. J. Pharm. 1994, 111 (3), 283–293. 10.1016/0378-5173(94)90351-4.

[ref146] Togias A. G.; et al. Demonstration of inhibition of mediator release from human mast cells by azatadine base: in vivo and in vitro evaluation. JAMA 1986, 255 (2), 225–229. 10.1001/jama.1986.03370020071030. 2416958

[ref147] Lichtenstein L. M.; Gillespie E. The effects of the H1 and H2 antihistamines on “allergic” histamine release and its inhibition by histamine. Journal of Pharmacology and Experimental Therapeutics 1975, 192 (2), 441–450. 46921

[ref148] Nadler M.; et al. Signal transduction by the high-affinity immunoglobulin E receptor FcRI: coupling form to function. Adv. Immunol. 2001, 76, 325–355. 10.1016/S0065-2776(01)76022-1. 11079101

[ref149] Kitani S.; et al. Inhibition of IgE-mediated histamine release by myosin light chain kinase inhibitors. Biochem. Biophys. Res. Commun. 1992, 183 (1), 48–54. 10.1016/0006-291X(92)91607-R. 1371921

[ref150] Hanifin J. M. The role of antihistamines in atopic dermatitis. J. Allergy Clin. Immunol. 1990, 86 (4), 666–669. 10.1016/S0091-6749(05)80237-4. 1699988

[ref151] Little M. M.; Casale T. B. Azelastine inhibits IgE-mediated human basophil histamine release. J. Allergy Clin. Immunol. 1989, 83 (5), 862–865. 10.1016/0091-6749(89)90096-1. 2469707

[ref152] Katayama S. Anti-allergic effect of azelastine hydrochloride on immediate type hypersensitivity reactions in vivo and in vitro. Arzneimittelforschung 1981, 31, 1196–1203. 6170298

[ref153] Chand N.; et al. Inhibition of IgE-mediated allergic histamine release from rat peritoneal mast cells by azelastine and selected antiallergic drugs. Agents Actions 1985, 16 (5), 318–322. 10.1007/BF01982866. 2413739

[ref154] Fischer B.; Schmutzler W. Inhibition by azelastine of the immunologically induced histamine release from isolated guinea pig mast cells. Arzneimittel-forschung 1981, 31 (8), 1193–1195. 6170297

[ref155] Michel L.; De Vos C.; Dubertret L. Cetirizine effects on the cutaneous allergic reaction in humans. Annals of allergy 1990, 65 (6), 512. 1979475

[ref156] Church M. K.; GRADIDGE C. F. Inhibition of histamine release from human lung in vitro by antihistamines and related drugs. British journal of pharmacology 1980, 69 (4), 663–667. 10.1111/j.1476-5381.1980.tb07919.x. 6159940 PMC2044314

[ref157] Vannieuwenhuyse E.; et al. Double-blind placebo-controlled clinical evaluation of oxatimide (R 35443). A novel potent anti-allergic drug in the treatment of hay fever. Allergy 1977, 32 (4), 278–289. 10.1111/j.1398-9995.1977.tb01359.x. 70947

[ref158] Clerck F.; et al. Oxatomide protectsTrichinella spiralis infected mice from lethal anaphylaxis. Agents Actions 1978, 8 (6), 568–571. 10.1007/BF01998884. 742554

[ref159] Seddon J. Inverse cubic phases of membrane-lipids, and their relevance to the static and dynamic structure of biomembranes. Acta Pharm. 1992, 42 (4), 255–262.

[ref160] Blijleven J. S.; Mechanisms of influenza viral membrane fusion. In Seminars in cell & developmental biology*;* Elsevier, 2016. 10.1016/j.semcdb.2016.07.007 27401120

