# Supplementary material for: Lipid Cubic Systems for Sustained and Controlled Delivery of Antihistamine Drugs
Source: Mol Pharm. 2021 Sep 22;18(10):3777–94. doi: 10.1021/acs.molpharmaceut.1c00279 (PMC8493555; doi:10.1021/acs.molpharmaceut.1c00279)
Supplement: Supplementary file 1 — mp1c00279_si_001.pdf [file mp1c00279_si_001.pdf]

Lipid cubic systems for sustained and controlled delivery of antihistamine drugs

Supplementary Information

Michele Dully[1], Miriama Ceresnakova[1], David Murray[2], Tewfik Soulimane[1],, Sarah P. Hudson[1],**

1 Department of Chemical Sciences, Bernal Institute, University of Limerick, Castletroy, Co. Limerick, Ireland

2 COOK Ireland Limited, O'Halloran Rd, Castletroy, Co. Limerick

*Corresponding author: E: sarah.hudson@ul.ie, tewfik.soulimane@ul.ie A: Bernal Institute, University of Limerick, Castletroy, Limerick, Ireland

Table S1. Properties, formulations and indications of the antihistamines investigated in this study

Antihistamine			Mol. weight (g/mol)	LogP value	BCS class*	Trade name	Indication	Dosage	Administration	Formulation
Decreasing aqueous solubility ⟶	1st generation	Diphenhydramine Hydrochloride (DPH)	291.8	3.65	I	Benadryl	Wide spectrum allergy, cough	6.25 - 50 mg orally every 4 to 6 hours [1]	Oral; sterile, pyrogen-free solution in water for injection for intramuscular or intravenous use [2]	Chewable or dissolvable tab; as a cream (containing propyl hydroxybenzoate, liquid paraffin, emulsifying wax and purified water); a gel (sometimes includes skin protectants such as allantoin, zinc acetate as well as inactive ingredients camphor, citric acid, ethanol, glycerin, methylparaben, propylene glycol, propylparaben, sodium citrate); or pill [3].
		Carbinoxamine maleate (CBX)	406.9	3.23	I	Arbinoxa; Karbinal ER	Allergic/ vasomotor rhinitis, conjuntivitis, urticaria, angioedema.	4 - 8 mg 3 to 4 times daily [4]	Oral	Drug-polistirex complex oral suspension under the trade name Karbinal ER [5], or as a tablet under trade name Arbinoxa [6]. The antihistamine is also formulated with decongestant pseudoephedrine HCl as oral treatment Rondec to treat hayfever, allergies and other breathing difficulties.
	2nd generation	Cetirizine Dihydrochloride (CZH)	461.89	2.98	III	Zyrtec	hay fever, allergic rhinitis, chronic urticaria and asthma	10 mg once daily [7]	Oral; Transdermal [8]	Formulated and supplied as a syrup, chewable tab or pill [3] but has also been explored for used in transdermal applications in the form of a cream containing propylene glycol as an emulsifying solvent [8].
		Azelastine Hydrochloride (AZL)	418.4	4.04	IV	Astelin; Asterpro	allergic rhinitis	0.28 mg once daily [9]	Nasal spray; eye drops	Racemic mixture intranasal spray [10]. Aqueous solution containing surfactant benzalkonium chloride [11].

*BCS is biopharmaceutics classification system

Calibration Curves

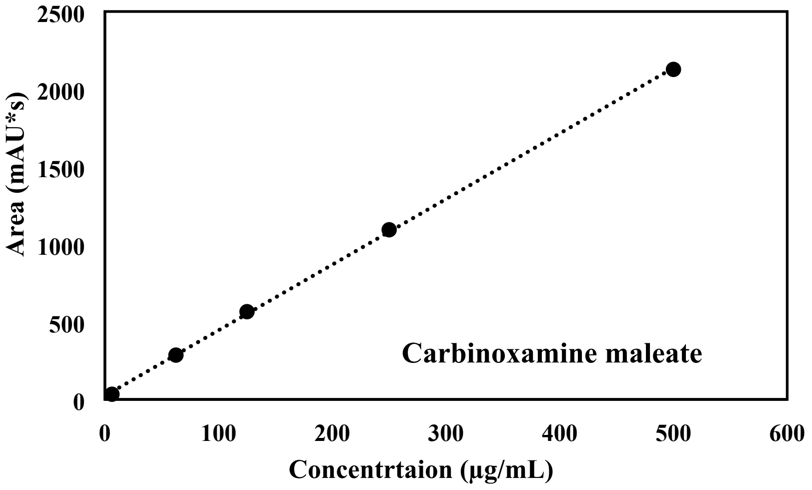

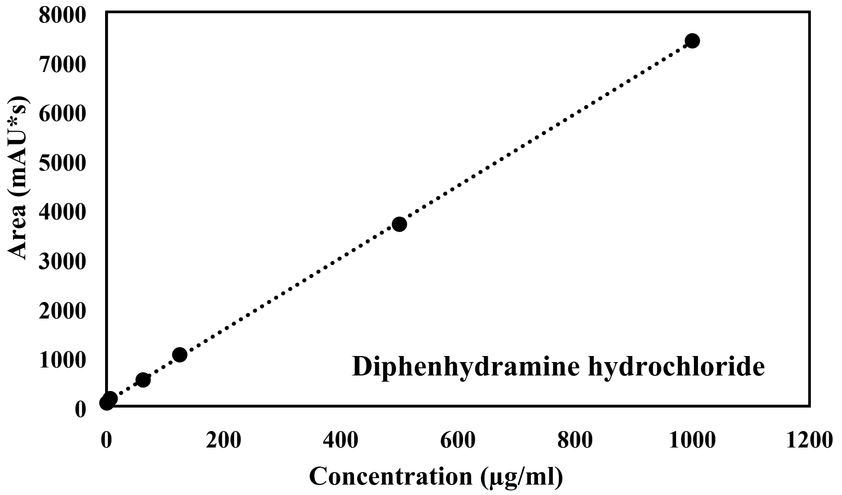

Figure S1. Calibration curves for 1st generation antihistamines Carbinoxamine maleate (CBX) and Diphenhydramine hydrochloride (DPH) obtained by HPLC

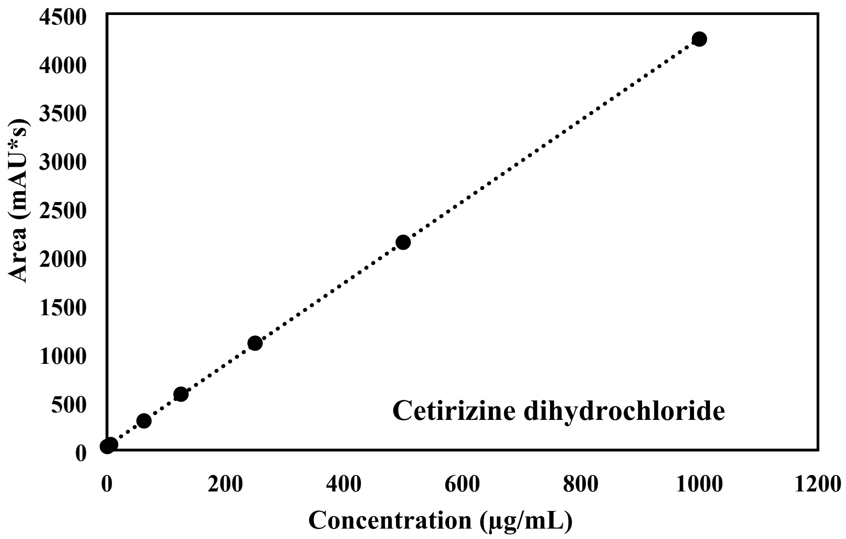

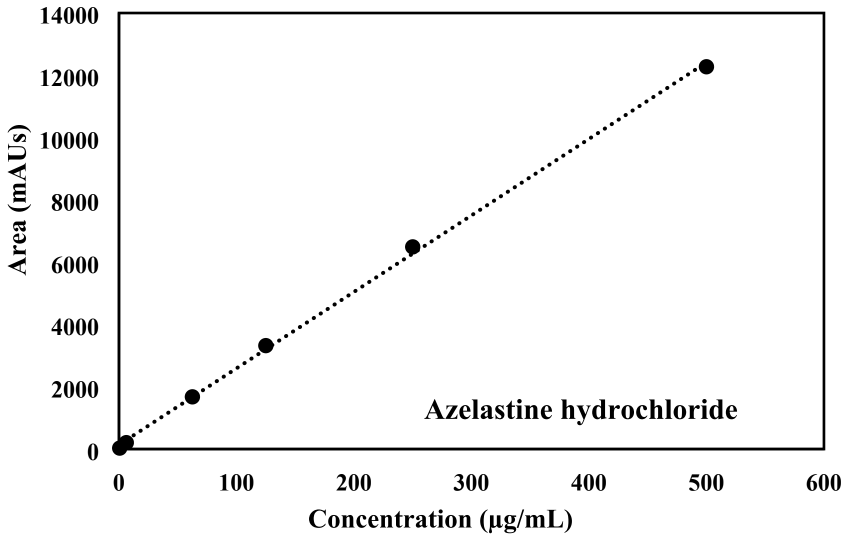

Figure S2. Calibration curves for 2nd generation antihistamines Cetirizine dihydrochloride (CZH) and Azelastine hydrochloride (AZL) obtained by HPLC

Mucoadhesion Studies: MP-SPR investigation

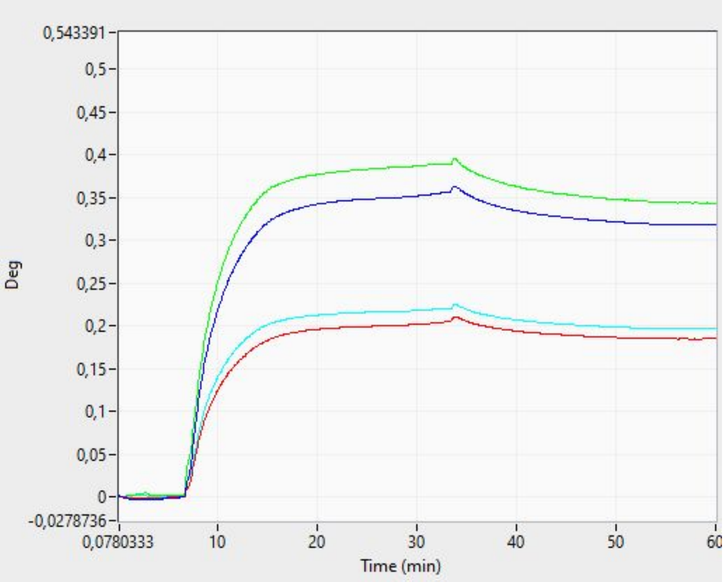

Figure S3. MP-SPR sensogram from adsorption of mucin onto clean Au sensor registered at two wavelengths (670 and 785 nm). Mucin (100 ug/ml in PBS) was injected into 2 channels at a flow rate of 30ul/min.

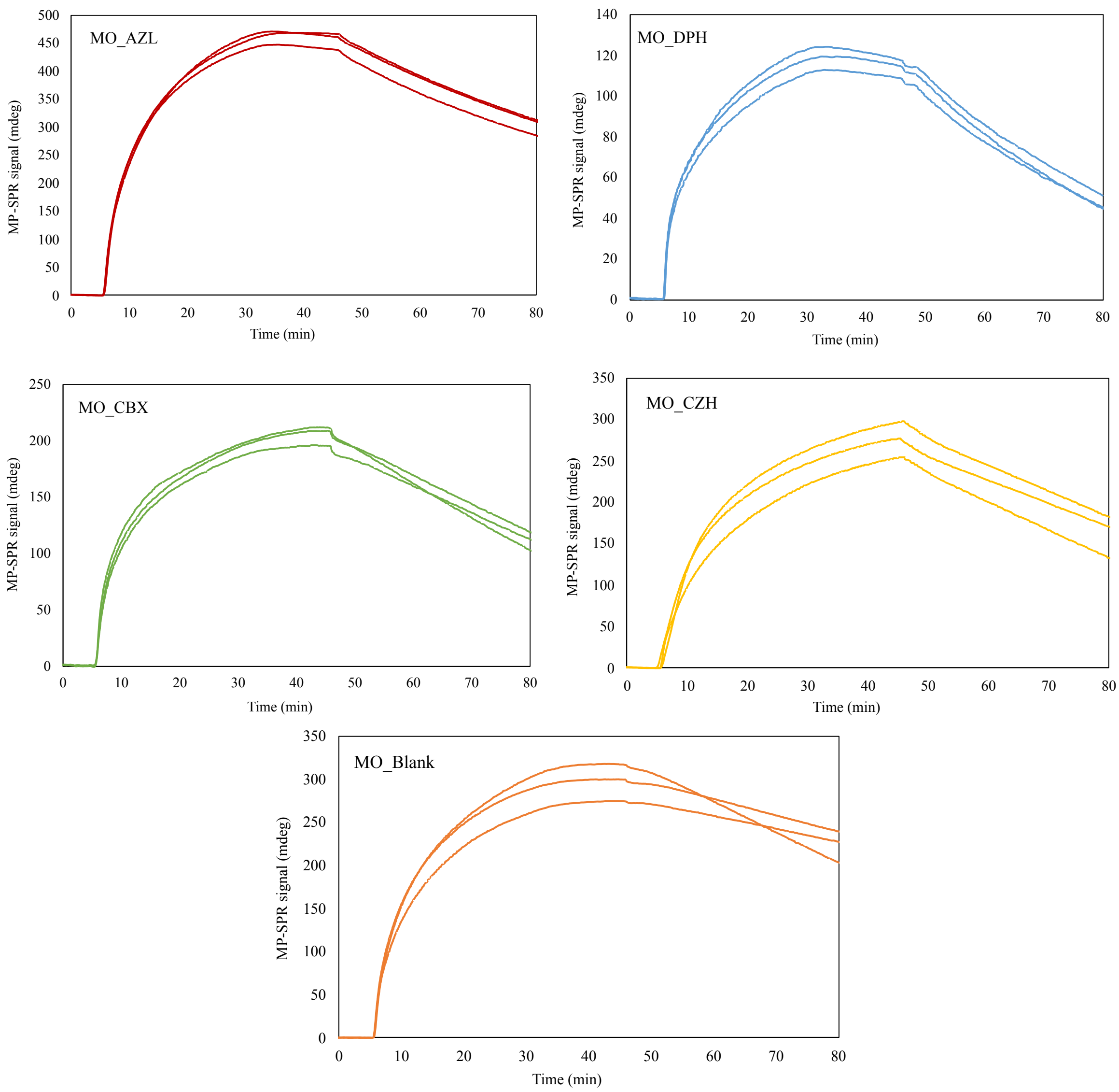

Figure S4. Kinetics of adsorption to mucin as measured by MP-SPR: overlay of triplicate injection for each tested lipid analyte.

PXRD of antihistamine drugs

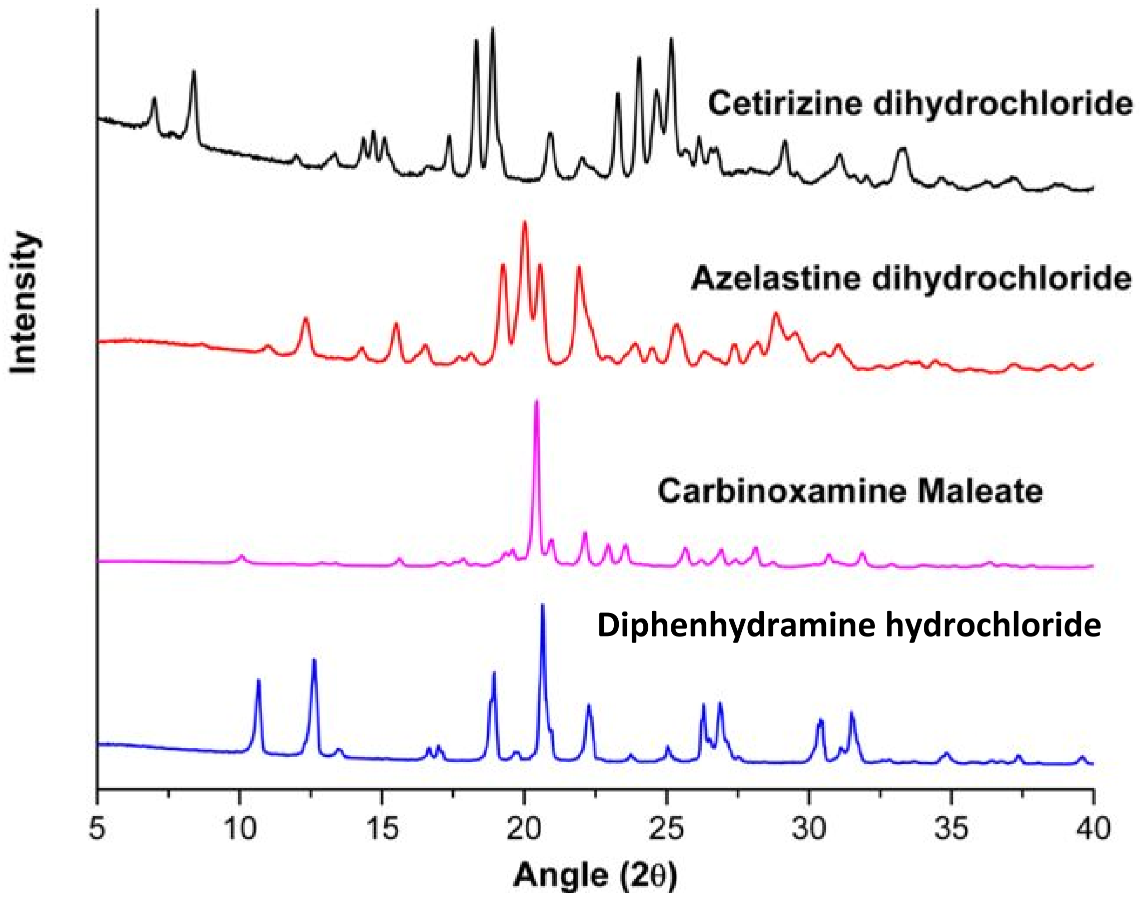

Figure S5. Diffractograms of the as-received antihistamine molecules

SAXS patterns of antihistamine-loaded LCP

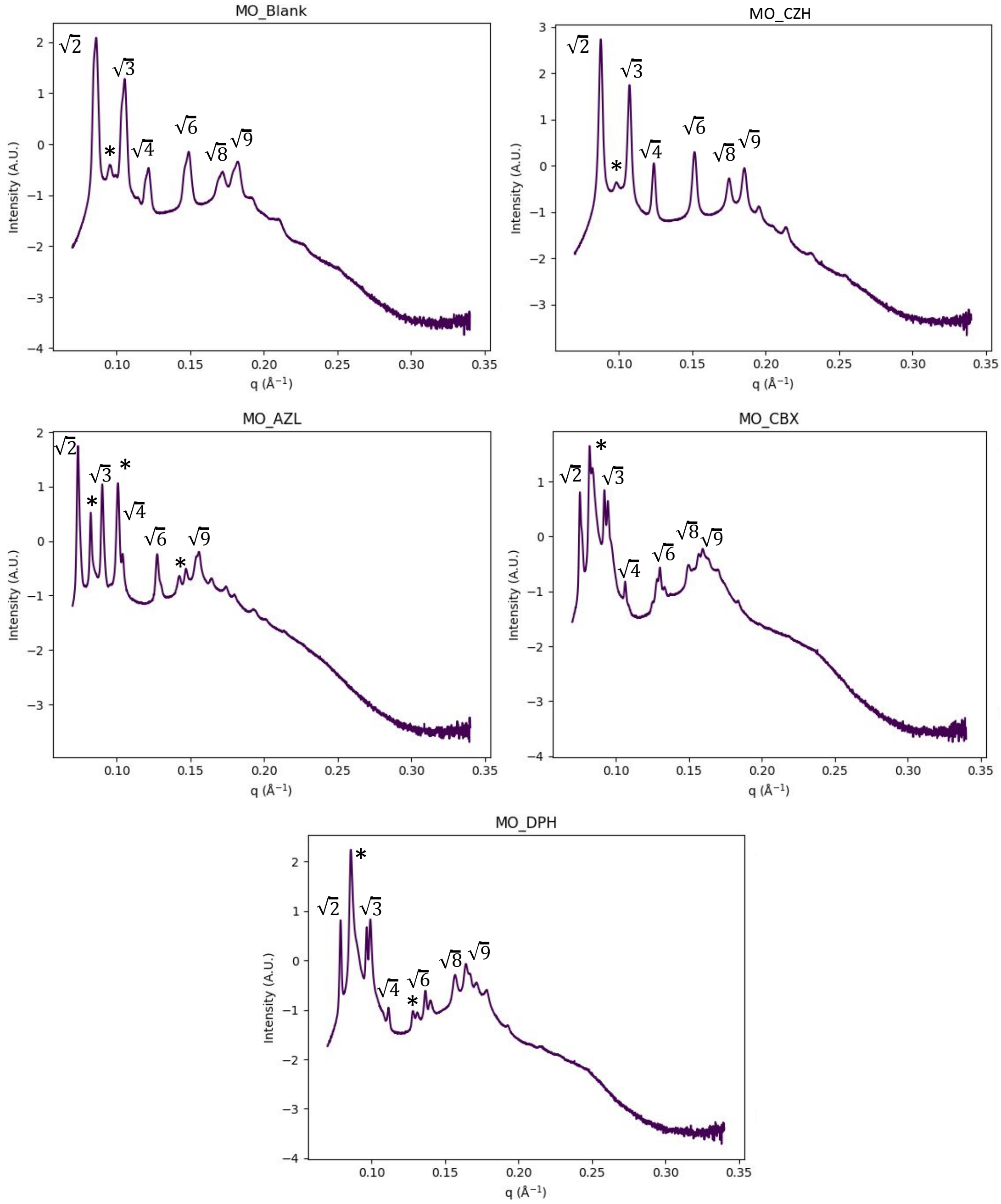

Figure S6. 1D azimuthally integrated SAXS patterns of bulk monoolein (MO) cubic mesophases formulated with or without antihistamines. The peaks of the patterns have been indexed according to the Q_{II}^{D} mesophase. Peaks that could not rationally be assigned to a mesophase are marked by an *.

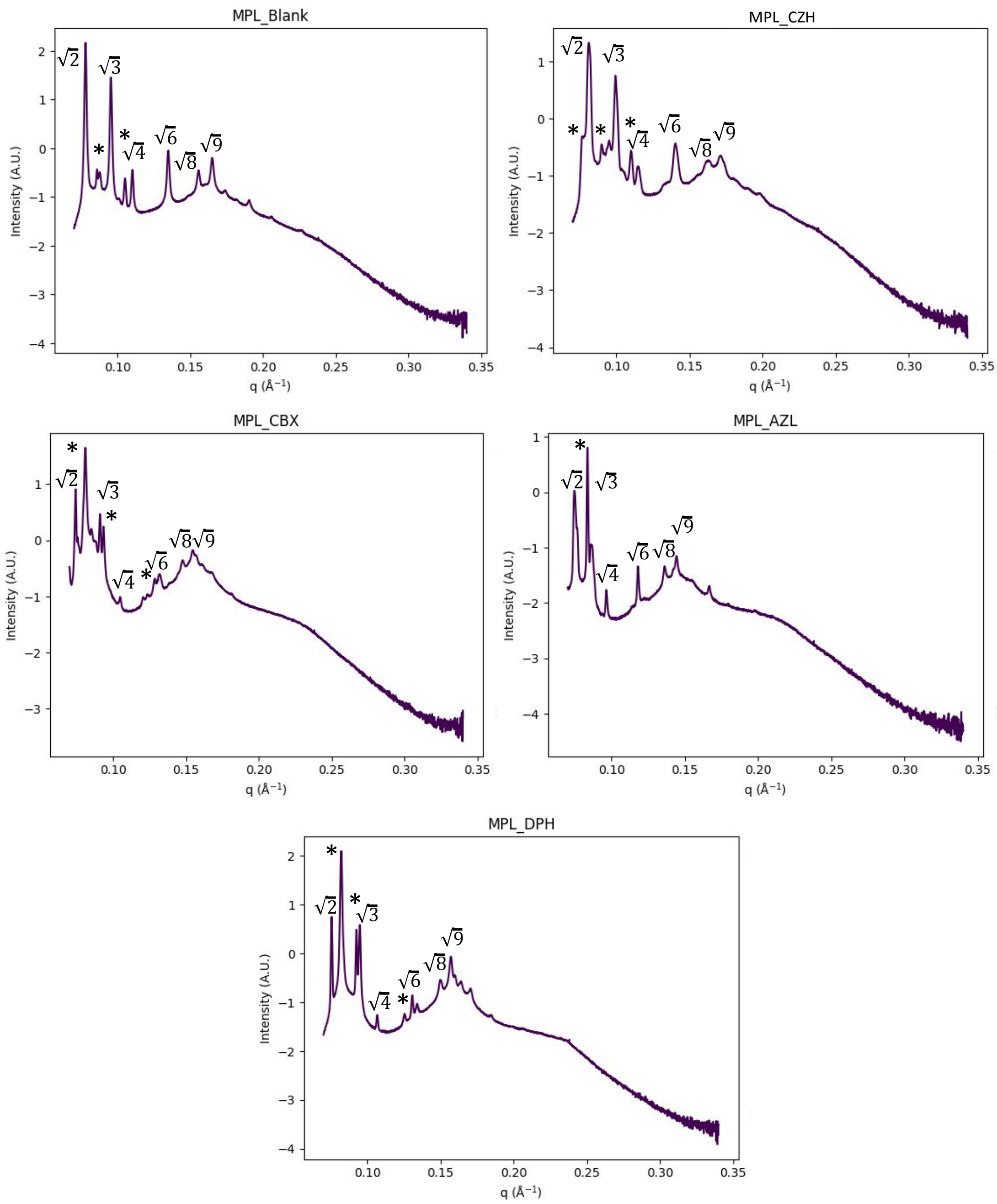

Figure S7. 1D azimuthally integrated SAXS patterns of bulk monopalmitolein (MPL) cubic mesophases formulated with or without antihistamines. The peaks of the patterns have been indexed according to the Q_{II}^{D} mesophase. Peaks that could not rationally be assigned to a mesophase are marked by an *.

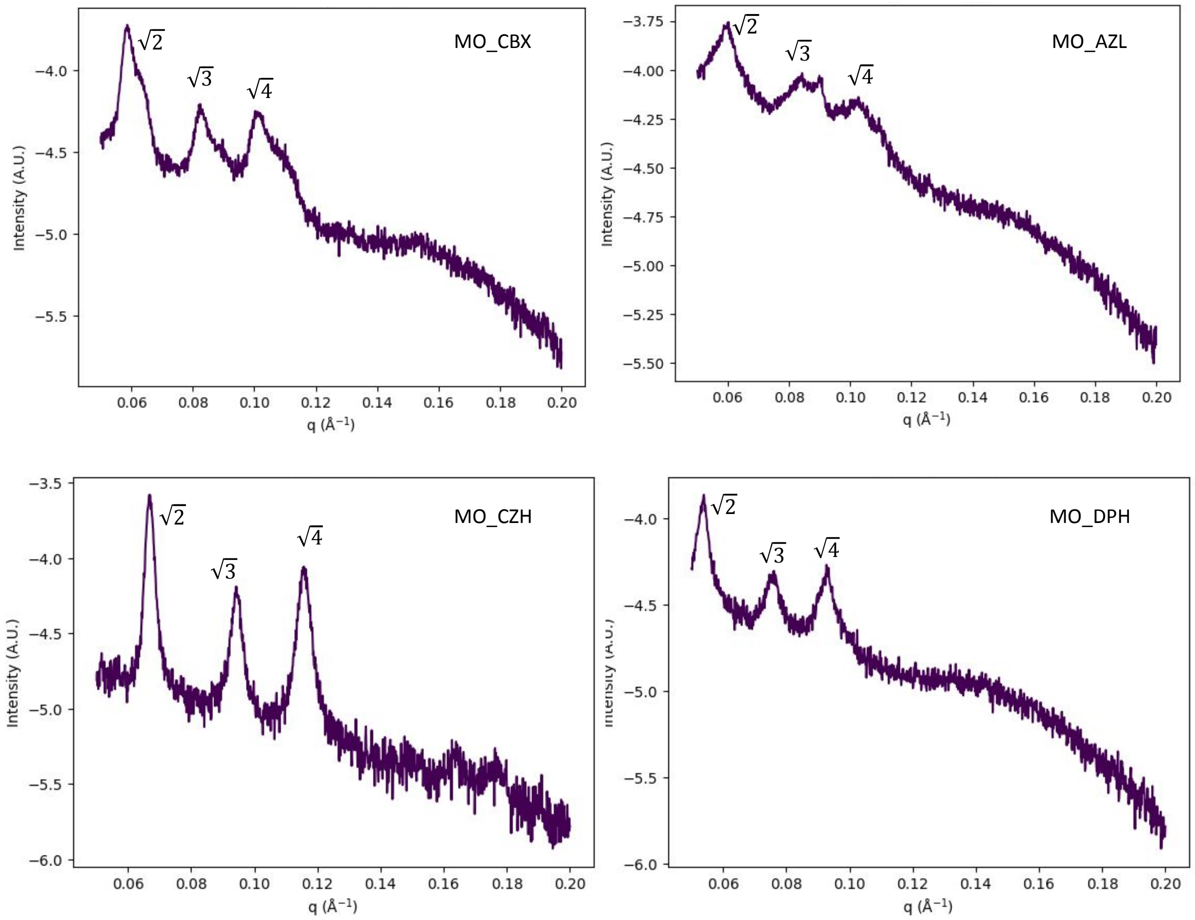

Figure S8. 1D azimuthally integrated SAXS patterns of monoolein (MO) cubosomes formulated with antihistamines. The peaks of the patterns have been indexed according to the Q_{II}^{D} mesophase.

References

1. Gelotte, C.K., B.A. Zimmerman, and G.A. Thompson, *Single-Dose Pharmacokinetic Study of Diphenhydramine HCl in Children and Adolescents.* Clinical pharmacology in drug development, 2018. **7**(4): p. 400-407.
2. BD Simplist, *Diphenhydramine Hydrochloride Injection, USP.* 2010.
3. Banerji, A., A.A. Long, and C.A. Camargo Jr. *Diphenhydramine versus nonsedating antihistamines for acute allergic reactions: A literature review*. in *Allergy & Asthma Proceedings*. 2007.
4. Edwards, R.J., C.-T. Huang, and N.-M. Pui, *Long acting dual release product containing carbinoxamine and pseudoephedrine*. 2016, Google Patents.
5. Food and Drug Administration, *HIGHLIGHTS OF PRESCRIBING INFORMATION: Karbinal ER*. 2013.

6. FDA. *Arbinoxa Oral Solution*. 2020; Available from: https://www.drugs.com/pro/arbinoxa-oral-solution.html.
7. Goindi, S., B. Dhatt, and A. Kaur, *Ethosomes-based topical delivery system of antihistaminic drug for treatment of skin allergies.* Journal of microencapsulation, 2014. **31**(7): p. 716-724.
8. Zemtsov, A. and H. Hosier, *A Novel Vehicle Formulation for Treatment of Inflammatory Skin Diseases.* Journal of Cosmetics, Dermatological Sciences and Applications, 2013. **3**(01): p. 18.
9. Salib, R.J. and P.H. Howarth, *Safety and tolerability profiles of intranasal antihistamines and intranasal corticosteroids in the treatment of allergic rhinitis.* Drug Safety, 2003. **26**(12): p. 863-893.
10. Hampel, F.C., et al., *Double-blind, placebo-controlled study of azelastine and fluticasone in a single nasal spray delivery device.* Annals of Allergy, Asthma & Immunology, 2010. **105**(2): p. 168-173.
11. Food and Drug Administration, *ASTELIN - azelastine hydrochloride spray, metered* 2011.
